# Human papillomavirus integration transforms chromatin to drive oncogenesis

**DOI:** 10.1186/s13059-023-02926-9

**Published:** 2023-06-27

**Authors:** Mehran Karimzadeh, Christopher Arlidge, Ariana Rostami, Mathieu Lupien, Scott V. Bratman, Michael M. Hoffman

**Affiliations:** 1 Department of Medical Biophysics, University of Toronto, Toronto, ON Canada; 2 Princess Margaret Cancer Centre, University Health Network, Toronto, ON Canada; 3 Vector Institute for Artificial Intelligence, Toronto, ON Canada; 4 Department of Computer Science, University of Toronto, Toronto, ON Canada

## Abstract

**Background:**

Human papillomavirus (HPV) drives almost all cervical cancers and up to 70% of head and neck cancers. Frequent integration into the host genome occurs predominantly in tumorigenic types of HPV. We hypothesize that changes in chromatin state at the location of integration can result in changes in gene expression that contribute to the tumorigenicity of HPV.

**Results:**

We find that viral integration events often occur along with changes in chromatin state and expression of genes near the integration site. We investigate whether introduction of new transcription factor binding sites due to HPV integration could invoke these changes. Some regions within the HPV genome, particularly the position of a conserved CTCF binding site, show enriched chromatin accessibility signal. ChIP-seq reveals that the conserved CTCF binding site within the HPV genome binds CTCF in 4 HPV^﻿+^ cancer cell lines. Significant changes in CTCF binding pattern and increases in chromatin accessibility occur exclusively within 100 kbp of HPV integration sites. The chromatin changes co-occur with out-sized changes in transcription and alternative splicing of local genes. Analysis of The Cancer Genome Atlas (TCGA) HPV^+^ tumors indicates that HPV integration upregulates genes which have significantly higher essentiality scores compared to randomly selected upregulated genes from the same tumors.

**Conclusions:**

Our results suggest that introduction of a new CTCF binding site due to HPV integration reorganizes chromatin state and upregulates genes essential for tumor viability in some HPV^+^ tumors. These findings emphasize a newly recognized role of HPV integration in oncogenesis.

**Supplementary Information:**

The online version contains supplementary material available at 10.1186/s13059-023-02926-9.

## Background

HPVs induce epithelial lesions ranging from warts to metastatic tumors [1, 2]. Of the more than 200 characterized HPV types [3], most share a common gene architecture [4]. As the most well-recognized HPV oncoproteins, E6 and E7 are essential for tumorigenesis in some HPV^+^ tumor models [5–7].

Beyond the oncogenic pathways driven by E6 and E7, emerging evidence suggests that high-risk HPV types play an important role in epigenomic regulation of tumorigenesis. While benign papillomas usually have episomal HPV [4], over 80% of HPV^+^ invasive cancers have integrated forms of HPV. Integration of HPV, therefore, results in a less favorable outcome [8]. Several studies indicate dysregulation of the transcriptome and epigenome upon integration [9–11]. Our knowledge of the mechanism and impact of this dysregulation, however, remains quite limited.

High-risk HPV types have a conserved binding site for the CTCF transcription factor [12]. CTCF binds to the episomal (circular and non-integrated) HPV at the position of this sequence motif and regulates the expression of E6 and E7 [12]. CTCF and YY1 interact by forming a loop which represses the expression of E6 and E7 in episomal HPV [13]. HPV integration may disrupt this loop and thereby lead to upregulated E6 and E7.

CTCF has well-established roles in regulating the 3D conformation of the human genome [14]. CTCF binding sites mark the boundaries of topological domains by blocking loop extrusion through the cohesin complex [15]. Mutations disrupting CTCF binding sites reorganize chromatin, potentially enabling tumorigenesis [16–18].

Introduction of a new CTCF binding site by HPV integration could have oncogenic reverberations beyond the transcription of E6 and E7, by affecting chromatin. Here, we investigate this scenario—examining how HPV integration in tumors results in local changes in the epigenome, gene expression, and alternative splicing—and propose new pathways to tumorigenesis driven by these changes.

## Results

### CTCF binds a conserved binding site in the host-integrated HPV

#### A specific CTCF sequence motif occurs more frequently in tumorigenic HPV types than any other motif

**Fig. 1:**
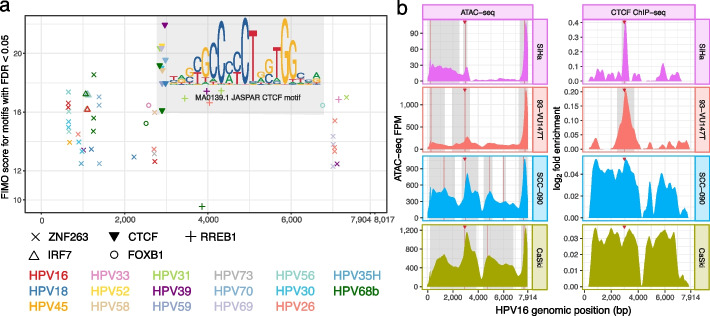
CTCF binds to its conserved binding site in HPV. **a** Transcription factor motif enrichment within the HPV genome. Horizontal axis: HPV genomic position (7904 bp for HPV16 and 8017 bp for the longest HPV genome among the 17). Points: Find Individual Motif Occurrences (FIMO) [26] enrichment scores of sequence motif matches ($q < 0.05$) of motifs occurring in at least 2/17 tumorigenic types; symbols: motifs; colors: HPV types. Gray shading: all shown matches for the CTCF motif and its sequence logo [27]. We showed the logo for the reverse complement of the JASPAR [24] CTCF motif (MA0139.1) to emphasize the CCCTC consensus sequence. **b** ATAC-seq MACS2 FPM (left) and CTCF ChIP-seq MACS2 $\log _{2}$ fold enrichment over the HPV16 genome (right) for 4 cell lines. To indicate no binding for regions with negative CTCF ChIP-seq $\log _{2}$ fold enrichment signal, we showed them as 0. Gray panels: MACS2 peak. Red vertical line: summit of MACS2 peak. Red triangle: position of the conserved CTCF sequence motif in HPV16. Dashed lines: HPV integration sites in each of the 4 cell lines 93-VU147T (orange), CaSki (moss), SCC-090 (blue), and SiHa (pink)

We searched the genome of tumorigenic HPV types for conserved transcription factor sequence motifs. Specifically, we examined 17 HPV types in TCGA head and neck squamous cell carcinoma (HNSC) [19] and cervical squamous cell carcinoma (CESC) [20] datasets [21]. According to the evaluation criteria of the International Agency for Research on Cancer Working Group on the Evaluation of Carcinogenic Risks to Humans [22], among these 17 types (Fig. 1a), 11 types show sufficient evidence for carcinogenicity. One type (HPV68b) shows limited evidence of carcinogenicity in humans but strong mechanistic evidence, three types (HPV26, HPV70, and HPV73) show limited evidence of carcinogenicity, and two types (HPV30 and HPV69) belong to the same species [23] as HPVs with sufficient or limited evidence of carcinogenicity. In each type’s genome, we calculated the enrichment of 518 JASPAR [24] transcription factor motifs (Fig. 1a). ZNF263 and CTCF motifs had significant enrichment at the same genomic regions within several tumorigenic types ($q < 0.05$). Only in CTCF motifs, however, did motif score enrichment in tumorigenic types exceed that of non-tumorigenic types (two-sample *t*-test $p = 0.02$; $t = -2.2$). The CTCF sequence motif at position 2916 of HPV16 occurred in the highest number of HPV types (10/17 types) compared to any other sequence motif (Fig. 1a). This position also overlapped with the strongest CTCF ChIP-seq signal observed in the uterine squamous cell carcinoma cell line SiHa [25] (Fig. 1a). The HPV16 match’s sequence TGGCACC**A**C**T**TGGTGGTTA closely resembled the consensus CTCF binding sequence [24], excepting two nucleotides written in bold ($p = 0.00001$; $q = 0.21$).

#### CTCF binds its conserved binding site in host-integrated HPV16

To test the function of the conserved CTCF motif in host-integrated HPV16, we performed ATAC-seq, CTCF ChIP-seq, and RNA-seq on 5 HPV16^+^ cell lines: 93-VU147T [28] (7 integration sites), CaSki [29] (6 integration sites), HMS-001 [30] (1 integration site), SCC-090 [31] (1 integration site), and SiHa [25] (2 integration sites). Unlike the other 4 cell lines, HMS-001 has only one incomplete integration of HPV into the host genome, and it lacks the genomic region containing the conserved CTCF motif [30]. For this reason, we only used HMS-001 within the comparison group. MACS2 identified a CTCF ChIP-seq peak within the HPV genomes of SiHa and 93-VU147T. In these 2 cell lines, the strongest CTCF ChIP-seq peak of the HPV genome aligned to the conserved CTCF sequence motif described above (Fig. 1b, right). In each of the 4 cell lines, the second-strongest chromatin accessibility peak aligned to both the CTCF sequence motif and the CTCF ChIP-seq peak (Fig. 1b, left).

The presence of both episomal and host-integrated HPV complicates the interpretation of HPV genomic signals. SiHa, however, does not contain episomal HPV [32, 33]. All of the ATAC-seq and RNA-seq SiHa fragments mapping to the integration site close to the conserved CTCF motif (HPV16:3,131) also partially mapped to the host genome at chr13:73,503,424. This also occurred for 3 of the 21 unpaired CTCF ChIP-seq reads mapping to HPV16:3,131. In agreement with previous reports [32, 33], these results suggest that the SiHa signal comes from the host-integrated HPV and that CTCF binding persists after HPV integration.

### HPV integration dysregulates chromatin accessibility and transcription

#### HPV dysregulates the local chromatin and transcriptome of a TCGA tumor

**Fig. 2:**
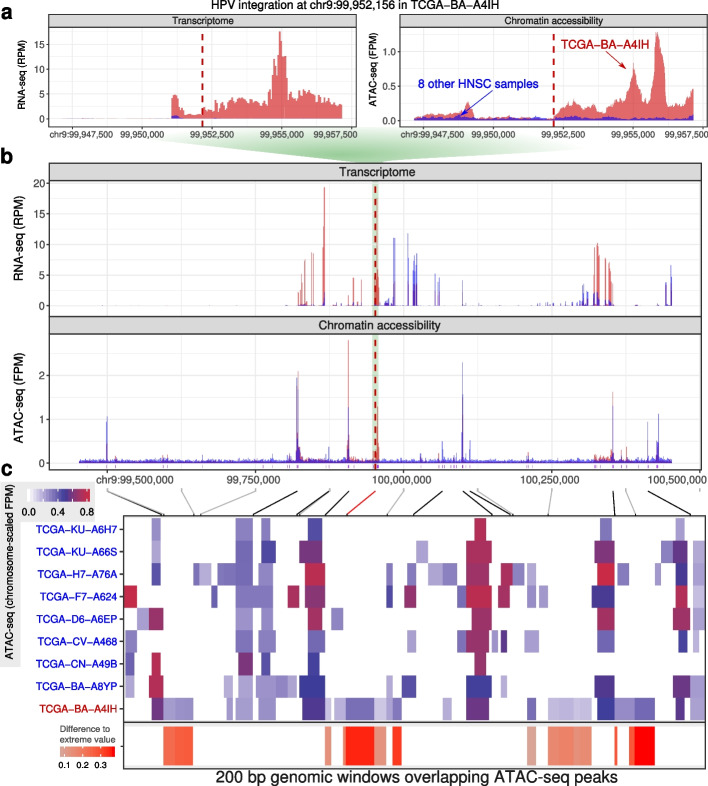
HPV integration alters the local transcriptome and epigenome. **a** A 10-kbp genomic window centered on TCGA-BA-A4IH’s HPV integration site. reads per million mapped reads (RPM) RNA expression (left); fragments per million (FPM) chromatin accessibility (right). Red: signal from TCGA-BA-A4IH; blue: signal from each of the 8 other HNSC samples. Vertical dashed red line: integration site. **b** Same data as (**a**), but in an expanded 1 Mbp genomic window. The green background shows how the coordinates of (**a**) fit in (**b**). The purple vertical bars show position of all ATAC-seq peaks found in any of the 9 tumor samples. **c** *(Top)*: Mapping of genomic positions for peaks with outlier signal in TCGA-BA-A4IH (gray), the position of the HPV integration site (red), and each 250,000 bp tick mark to ATAC-seq peaks. Gray diagonal lines map each 250,000 bp to the corresponding peaks. The black lines map the genomic position of the top 9 peaks with the strongest FPM in any of the 9 samples to the corresponding peaks. *(Middle)*: Heatmap of ATAC-seq peaks in the same 1 Mbp genomic window. Color indicates ATAC-seq FPM divided by the maximum FPM value of chr9 in each patient (see the “﻿ATAC-seq” section). Each column shows a 200-bp genomic window overlapping a peak in any of the 9 patients. We showed all 200-bp genomic windows with sliding windows of 50 bp if the window overlaps a peak. *(Bottom)*: Difference of the values in TCGA-BA-A4IH and the most extreme value in the other 8 patients when TCGA-BA-A4IH had the most extreme value among the 9 patients. We used white when TCGA-BA-A4IH did not have the most extreme value

As observed in SiHa, integration of HPV into the host genome generates *chimeric* sequences evidenced by sequencing reads that partially map to both the host and viral genomes. We characterized high-confidence HPV integration sites containing chimeric sequences from TCGA cases (see the “Identifying HPV integration sites” section; Additional file 1﻿: Table S1). Nine TCGA HNSC patients have both matched RNA-seq data measured in reads per million mapped reads (RPM) and ATAC-seq data measured in fragments per million (FPM) [34]. Using the RNA-seq data, we identified an HPV integration site in TCGA-BA-A4IH at chr9:99,952,156. The transcriptome and chromatin accessibility of this patient differed greatly from the other 8 patients at the HPV integration site (Fig. 2). The other 8 patients lacked transcription ($\text {RPM} < 1$) or chromatin accessibility ($\text {FPM} < 0.2$) within 5 kbp of the integration site. TCGA-BA-A4IH, however, exhibited both active transcription and accessible chromatin (Fig. 2a). In fact, TCGA-BA-A4IH’s chromatin accessibility and RNA expression exceeded the other 8 patients up to 400 kbp beyond the integration site (Fig. 2b). Within those bounds, TCGA-BA-A4IH’s chromatin accessibility peaks often had signal exceeding that of all 8 other patients (Fig. 2c).

#### HPV dysregulates local chromatin and transcriptome in HPV^+^ cell lines

**Fig. 3:**
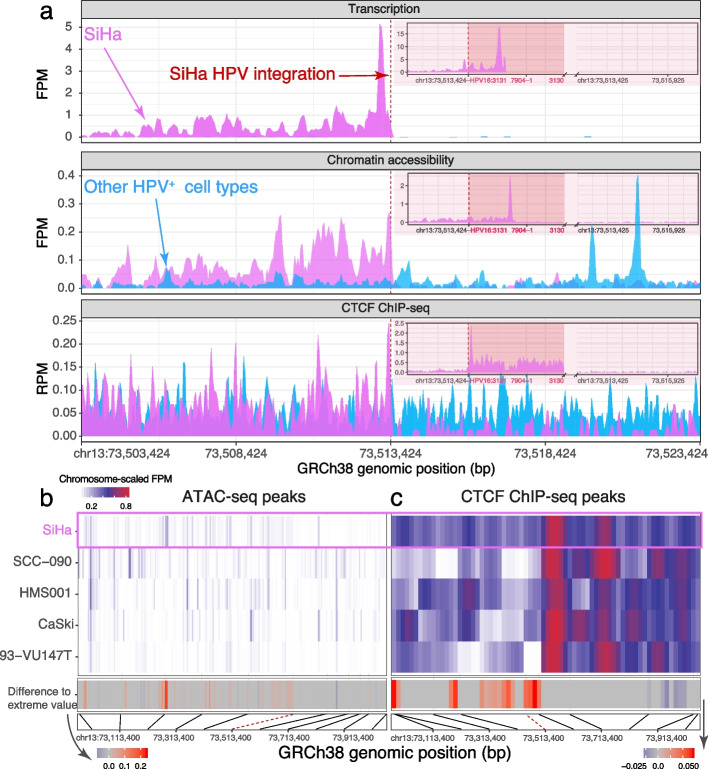
HPV integration disrupts local host epigenome and transcriptome. **a** Genomic assay signal for an HPV integration site of SiHa (chr13:73,513,424). Top: RNA expression FPM; middle: ATAC-seq FPM; bottom: CTCF ChIP-seq RPM. Pink bars: signal from SiHa; blue bars: signal from 4 other HPV^+^ cell lines without integration at this position. Red dashed line: HPV integration site. Inset at top right of each panel shows signal within the hybrid genomic window including the host genome upstream of the integration site, full-length HPV16 (red shading) beginning at the integration site, a gap representing uncertainty in the end of the integrated HPV genome, and the host genome downstream of the integration site. **b** ATAC-seq peaks in a 1-Mbp window centered on SiHa’s integration site. Each column shows a 200-bp genomic window overlapping a peak. We generated all 200-bp genomic windows with a stride of 50 bp which overlapped a peak in any of the 5 cell lines. *(Top)*: ATAC-seq FPM and CTCF ChIP-seq $\log _{2}$ fold enrichment over control for each cell line divided by the cell line’s corresponding maximum value in chromosome 13. *(Middle)*: Difference in the epigenome of SiHa and the most extreme value in the other 4 cell lines when SiHa had the most extreme value among the cell lines. When SiHa did not have the most extreme value, we used white. *(Bottom)*: Physical location of peaks. Black lines map every 100 kbp to the corresponding peak. Red dashed line: HPV integration site. **c** Similar to (**b**), but for CTCF ChIP-seq instead of ATAC-seq

To investigate the generalizability of the dysregulated chromatin and transcription that we observed in TCGA-BA-A4IH, we conducted a similar analysis on 5 HPV^+^ lines. For each HPV integration site, we compared the cell line with integrated HPV to the other 4 cell lines without HPV at that genomic position. Only the cell line with HPV integration displayed strong expression of nearby genes (Fig. 3a, top).

For each viral integration site, expression of the chimeric transcript occurred either only upstream (for 3 of the integration sites of 93-VU147T and 2 of the integration sites of CaSki) or only downstream (the other 12 integration sites), never in both directions (Fig. 2a). Directional chimeric transcription suggests that only one end of the integrated virus drove expression that continued past the integration site into the host genome.

Since we identified the integration site by detecting chimeric transcripts in RNA-seq data, we expected to observe transcription of the host genome at the site of viral integration. Nevertheless, transcription of these regions necessitates an active viral-dependent mechanism, as they are not transcribed in cell types without HPV integration in the same genomic regions (Fig. 3a, top). Among all HPV integration sites, expression of the viral-host chimera co-occurred with chromatin accessibility signal (Fig. 3a, middle). The overlap of transcription and chromatin accessibility suggests that viral integration introduces *cis*-regulatory elements which actively transcribe the viral-host chimera. The consistent recruitment of CTCF at HPV integration sites in 4 different cell lines and altered CTCF binding around integration sites suggest that CTCF might play a role in integration-dependent HPV tumorigenesis (Fig. 3a, bottom).

To understand the spatial effect of HPV integration on chromatin, we examined CTCF ChIP-seq and chromatin accessibility peaks in SiHa within 500 kbp of its chr13:73,513,424 integration site (Fig. 3b–c). Some of the regions of inaccessible chromatin in 93-VU147T, CaSki, and SCC-090 are accessible in SiHa within 500 kbp of this integration site. In many of these regions, SiHa had more accessible chromatin compared to any of the other 4 cell lines (Fig. 3b, middle). For CTCF, however, some genomic regions showed enrichment and other genomic regions showed depletion in CTCF binding (Fig. 3c).

**Fig. 4:**
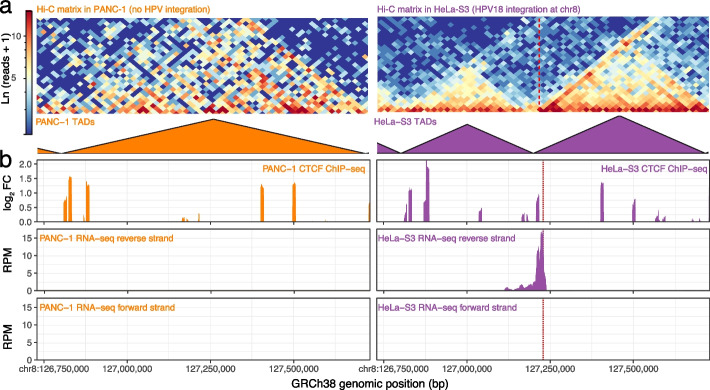
HPV integration forms new TAD boundaries in HeLa-S3. **a** PANC-1 (left; orange) and HeLa-S3 (right; purple) Hi-C matrices (top) and TAD boundaries (bottom) within a 1-Mbp genomic region centered at the HPV integration site of HeLa-S3 in chr8 (dashed red line in HeLa-S3 only). **b** CTCF ChIP-seq signal $\log _{2}$ fold enrichment over control (top), reverse strand RNA-seq signal RPM (middle), and forward strand RNA-seq signal RPM (bottom) in PANC-1 (left; orange) and HeLa-S3 (right; purple)

To investigate the possibility of topologically associating domain (TAD) re-organization, we used the 3D Genome Browser [35] to compare TAD boundaries of HeLa-S3 [36], a cervical carcinoma cell line with HPV integration, to other cell lines without HPV (PANC-1 [37], MCF-10A [38], GM12878 [39], K562 [40], and H1-hESC [41]). HeLa-S3 cells contained chromatin interactions, TADs, and a TAD boundary not found in other cell types (Fig. 4a–b). CTCF binding and RNA transcription increased in HeLa-S3 at the TAD boundary (Fig. 4c). While little transcription occurred at this genomic region in PANC-1, K562, GM12878, and H1-hESC, strand-specific transcription data indicated more transcription from the reverse strand in HeLa-S3 (Fig. 4c).

### Integration of HPV dysregulates expression and alternative splicing of local genes

#### HPV integration alters gene expression in HPV^+^ cell types

To determine further whether HPV integration significantly changed gene expression, we next examined changes in transcription of individual genes, as measured in transcripts per million (TPM), within HPV^+^ cell lines. We used two criteria to identify outlier changes in gene expression which occurred due to HPV integration. First, we calculated expression fold change dividing $\log _2 \text {TPM}$ in the sample with HPV integrated at some locus ($\text{TPM}_{\text {HPV}^{+}}$) by median TPM in samples without HPV integrated at that locus ($\langle \text{TPM}_{\text {other}}\rangle$). For an HPV^+^ cell line, we only considered a gene an outlier if its expression fold change exceeded 2 (see the “Identifying HPV-induced outlier expression” section).

**Fig. 5:**
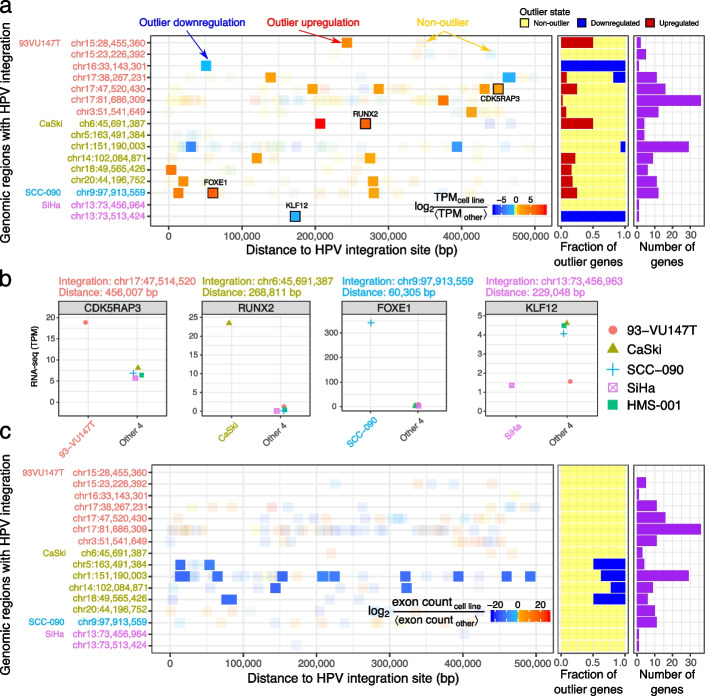
HPV integration alters local transcription and splicing. **a** (*Left*) Distances between 166 RefSeq genes within 500 kbp of 16 HPV integration sites. Color: $\log _2 \text {TPM}$ of the cell line with HPV integration divided by median TPM of the other 4 cell lines. Solid squares: 20 upregulated (red) and 4 downregulated (blue) outlier genes. Transparent squares: 140 genes without outlier change in gene expression. (*Middle*): Fraction of genes within 500 kbp of each HPV integration site which are either non-outlier (yellow), downregulated (blue), or upregulated (red). We labeled one gene from each cell type and visualized their TPM in (**b**). (*Right*): Number of genes within 500 kbp of each HPV integration site. For the overlapping integration sites in SiHa, we showed each gene in only one row to avoid duplication. **b** Expression of one outlier gene from each of the 5 cell lines compared to the other 4 cell lines without HPV around the gene. **c** Similar to (**a**) but for differential exon usage of 159 Ensembl genes within 500kbp of 16 HPV integration sites. Color: DEXSeq model fold change in exon count for the exon with the most extreme change in expression. Solid squares: 19 genes with DEXSeq $q < 0.2$ and absolute exon fold change $> 1$. Transparent squares: 140 genes without outlier change in exon usage

Out of the 16 HPV integration sites, 9 had upregulated genes only (expression fold change $> 2$), 3 had downregulated genes only (expression fold change $< -2$), and 1 (chr17:38,267,231 of 93-VU147T) had both upregulated and downregulated genes (Fig. 5a, middle).

#### HPV integration sites alter gene splicing

Our results suggested that HPV integration increases chromatin accessibility and alters CTCF binding. Since chromatin-binding proteins, including CTCF, can modify gene splicing [42], we investigated whether HPV integration affects alternative splicing of nearby genes.

We quantified how the expression of each exon varies independent of the global expression of that gene (see the “RNA-seq” section). HPV integration sites in CaSki displayed outlier expression of specific exons of genes within 500 kbp (Fig. 5c). Although perturbations in splicing regulators occur frequently in many cancer types [43], global dysregulation of splicing regulators within CaSki could not easily explain our findings. For example, the expression of genes involved in splicing regulation (16 biological processes containing the phrases “splicing”, “splice site”, or “spliceosome” in Gene Ontology (GO) v6.2) did not suggest a global change differentiating CaSki from the other 4 cell types. In addition, while 43/378 expressed genes involved in splicing had their highest expression in CaSki, other cell types also exhibited higher expression of other genes involved in splicing. Taken together, these results indicate that HPV integration can influence differential exon usage of neighboring genes.

### HPV modifies the epigenome and transcriptome within 100 kbp of integration sites

The dysregulation of gene expression and splicing near HPV integration sites may relate to altered chromatin variants. We investigated transcriptomic and epigenomic dysregulation upon HPV integration in the RNA-seq, ATAC-seq, and CTCF ChIP-seq data. At each integration site, we compared the genomic coverage of each assay for the cell line with HPV integration to the average in the other four cell lines:

1 $$\begin{aligned} \log _2 \frac{\text {RPM}_{\text {HPV}}}{\langle \text {RPM}_{\text {other}} \rangle } \text {.} \end{aligned}$$

This allowed us to distinguish sample-specific variability from variations due to HPV integration.

We calculated RPM fold change (Eq. 1) for all 10-kbp genomic windows around any HPV integration site. We calculated the same measurement for 10 random permutations of HPV integration sites. For each permutation, we moved the location of each HPV integration site in each cell line to a random integration site from another cell line, without replacement. We scrambled only the locations of the integration sites, leaving the assay data the same.

**Fig. 6:**
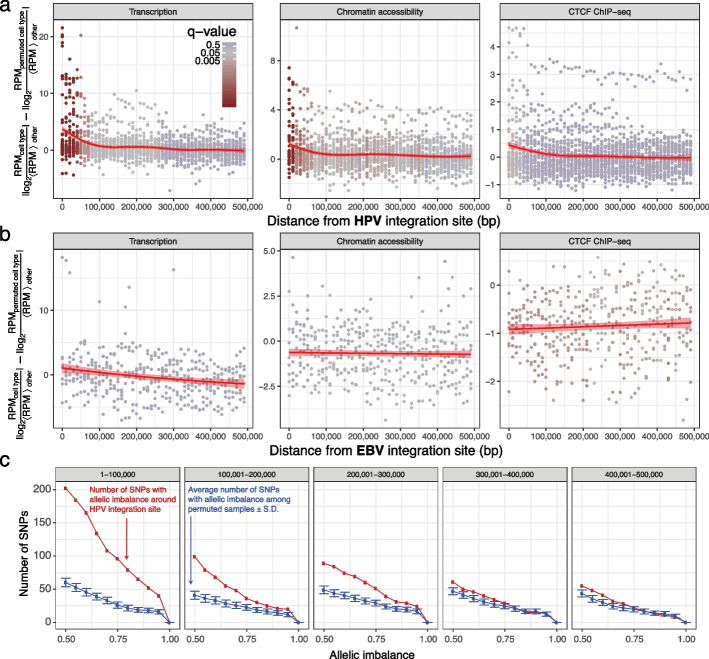
HPV integration dysregulates the epigenome and transcriptome up to 100 kbp away. **a** Difference in average RPM fold change in cell lines with HPV compared to 10 permuted controls. Each data point assesses the difference at a 10-kbp genomic bin. Color: *q*-value of *t*-test comparing the cell line with HPV integration to 10 permutations. Line: generalized additive model [44] regression model on transcription data (RNA-seq; left), chromatin accessibility (ATAC-seq; middle), and CTCF presence (ChIP-seq; right). **b** Same as (**a**) but comparing GM12878 Epstein-Barr virus (EBV) integration sites to 2 other lymphoblastoid cell lines. **c** Complementary cumulative distribution function of number of single nucleotide polymorphisms (SNPs) with allelic imbalance exceeding some threshold within some distance from each HPV integration site. Shown either for the sample with the HPV integration site (red) or the mean in 100 different permutations where we assigned each SNP to a cell type without HPV integration at that genomic position (blue). Error bar indicates ± standard deviation (SD). The 5 facets indicate the distance of SNPs from the HPV integration sites in in base pairs

For each assay, we examined the fold change of the original RPM against other cell types and compared with the fold change of the permuted RPM against other cell types. We did this for each 10-kbp window from the site of HPV integration up to 500 kbp away. We conducted a two-sided *t*-test on the differences (Fig. 6a).

RNA-seq, ATAC-seq, and CTCF ChIP-seq significantly differed between the original and permuted measurements up to 100 kbp from the HPV integration sites ($q < 0.05$). HPV’s effect size on transcription, chromatin accessibility, and CTCF binding diminished as distance from the HPV integration sites increased (Fig. 6a).

We hypothesized that changes in epigenome and transcriptome occurred due to a specific feature of the integrated HPV, and would not just arise from any genomic insertion. Under this hypothesis, we expected that the integration of the 170-kbp Epstein-Barr virus (EBV) genome would fail to induce similar changes to the 8-kbp HPV genome. EBV, unlike HPV, shows a significant enrichment in association with gene-depleted chromosomes, often through the 2-kbp genomic region *OriP* [45]. *OriP* does not show a significant enrichment for cohesin CTCF binding sites, suggesting a potentially different mechanism of viral-host interaction than HPV [45]. Therefore, we investigated how the transcriptome and epigenome changed at the EBV integration sites of 4 lymphoblastoid cell lines: GM12873, GM12878, GM23248, and GM23338 (Fig. 6b; Additional file 1: Table S2).

Unlike with HPV, we detected no significant difference in transcriptome or epigenome within 100 kbp of EBV integration sites (Fig. 6b). We observed more transcription around EBV integration sites, but no statistically significant difference after correcting for multiple comparisons ($q > 0.37$). GM12878 had less accessible chromatin and less CTCF binding compared to the other 2 lymphoblastoid cell lines when considering a larger region up to 500 kbp around EBV integration sites ($q < 0.05$). The magnitude of change, however, was relatively modest (RPM fold change of as much as $-4$) compared with the corresponding difference near HPV integration sites (RPM fold change of as much as 22).

To investigate whether these changes occurred in the chromosome harboring integrated HPV sequence, rather than a homologous chromosome without them, we examined allele-specific expression close to the HPV integration site. If the HPV integration itself caused increased expression, we should observe higher allele-specific expression, as quantified by allelic imbalance. We defined allelic imbalance as the fraction of reads corresponding to the more-expressed allele for each SNP according to dbSNP v147 [46]. Allelic imbalance, therefore, ranges from 0.5 to 1. To avoid rare base-calling errors affecting the analysis, we only examined SNPs with a minimum coverage of 10 reads and at least 3 reads supporting each of the reference and altered alleles.

Genes near integration sites had higher allele-specific expression. Higher allele-specific expression occurs particularly within 300 kbp of HPV integration in the sample with HPV integration, as compared to 100 permutations where we randomly assigned each allelic imbalance to a sample without HPV integration at that genomic position (Fig. 6c). These results further confirm that downstream transcriptional changes occur on the same chromosome containing HPV sequence.

#### HPV integration dysregulates the local transcriptome of HPV^+^ carcinomas

**Fig. 7:**
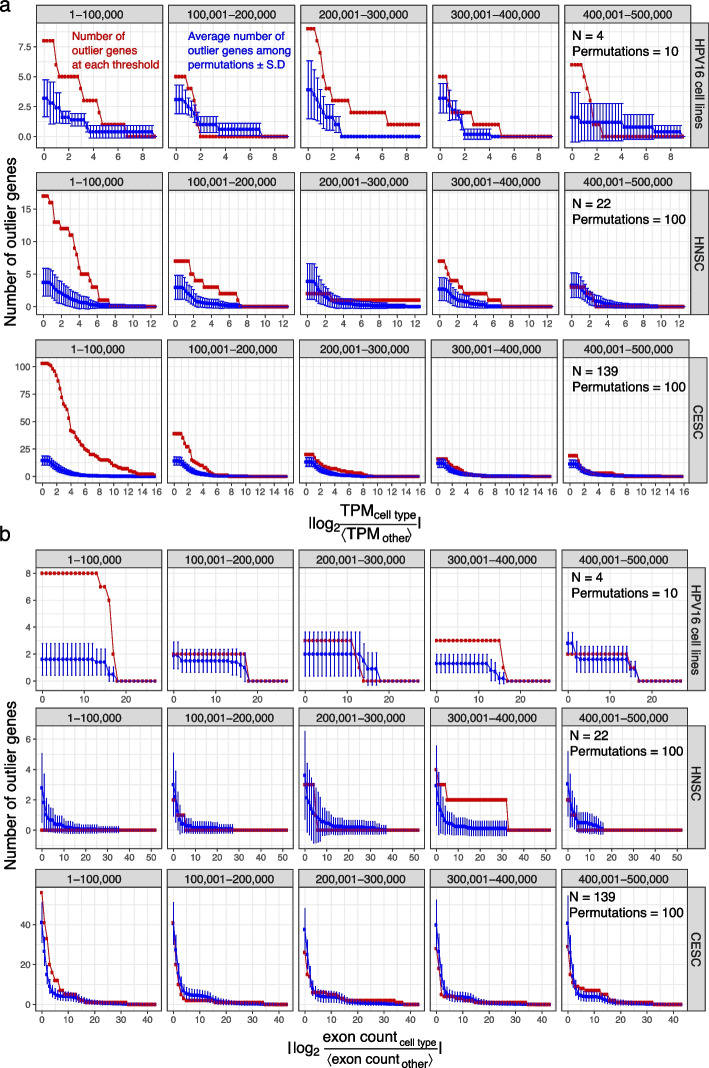
HPV integration dysregulates the local transcriptome. **a** Complementary cumulative distribution function of number of outlier genes exceeding plotted absolute expression fold change $\ge$ horizontal axis values and $\left\lvert{\text {TPM}_{\text {HPV}} - \left\langle \text {TPM}_{\text {other}} \right\rangle }\right\rvert$ exceeding twice the SD. Top: 5 HPV16^+^ cell lines. Middle: HNSC patients. Bottom: CESC patients. Red: number of outlier genes in RNA-seq data; blue: mean number of outlier genes in 10 permutations of the samples; error bars: SD. **b** Similar to (**a**), but each showing the number of genes with absolute fold change in exon count $> 1$ and DEXSeq $q < 0.2$

Both cell lines derived from HNSC (93-VU147T and SCC-090) and cell lines derived from CESC (CaSki and SiHa) displayed epigenomic and transcriptomic changes near HPV integration sites. To investigate how often outlier gene expression occurs due to biological variation other than HPV integration, we permuted RNA-seq data for these 4 cell lines, for TCGA HNSC samples, and for TCGA CESC samples. For HNSC and CESC datasets, we generated 100 permutations of samples as the background. For the 4 cell lines, however, we generated 10 permutations to avoid over-representation of the effects from the cell types with fewer viral integrations. In both the three original datasets and in corresponding permuted datasets each, we examined genes at several thresholds of expression fold change separated by intervals of 0.25. We identified those genes with expression exceeding each threshold where the difference $\left\lvert {\text{TPM}_\text{HPV} - \left\langle \text{TPM}_{\text {other}} \right\rangle } \right\rvert$ exceeded twice the SD (Fig. 7a).

The original datasets contained more outlier genes passing a fold change cutoff of 2 compared to the permuted controls. The greatest deviation of the original datasets compared to the permuted datasets occurred within the 100-kbp window of HPV integration. In the 4 cell lines examined, we detected 8 outlier genes within 100 kbp of HPV integration, but a mean of 5 outlier genes in the 10 permuted datasets. Among HNSC tumors, we identified 16 outlier genes, far greater than the mean of 2 outlier genes in the permuted HNSC controls. We also identified 103 outlier genes among CESC tumors—as opposed to a mean of 13 outlier genes within the permuted CESC controls.

We performed a similar permutation analysis to investigate whether differential exon usage occurs due to biological variations other than HPV integration (Fig. 7b). Within 100 kbp of HPV integration, we consistently identified more genes with differential exon usage in the original datasets compared to permuted controls. In the 5 cell lines examined, we found 8 genes with differential exon usage, but only a mean of 2 genes with differential exon usage among the permuted controls. In these 8 genes, absolute $\log _2$ exon count fold change (Eq. 2) exceeded 13 ($q < 0.2$). We found similar results for CESC tumors.

We investigated whether the direction of the chimeric HPV transcript affects the magnitude of changes in neighboring genomic regions. Although the most highly upregulated genes occurred near the HPV integration sites that induced the chimeric transcript downstream of the integration site, the association did not pass the statistical significance cutoff (linear model $p = 0.21$).

### HPV integration upregulates putative oncogenes

**Fig. 8:**
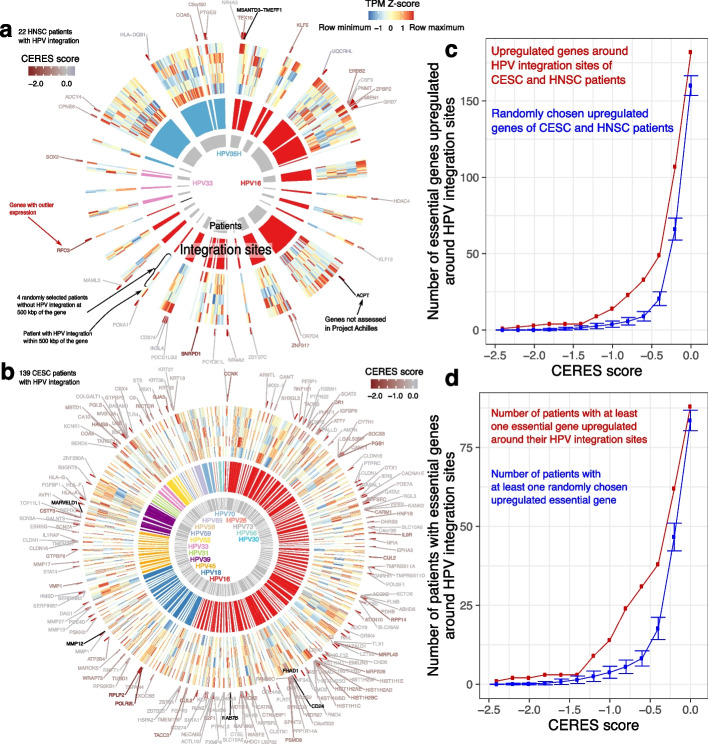
Outlier gene expression in HPV^+^ patients. **a** 26 TCGA HNSC patients with 3 types integrated at 35 sites. Inner gray ring: each arc indicates a patient. Middle ring: individual HPV integration sites, with color representing HPV type. Outer ring: heatmap of expression of genes within 500 kbp of each integration site in the patient with HPV integration (peripheral) and 4 randomly selected patients without HPV integration around that gene (central). Values show *Z*-score of TPM values of each gene among the 5 samples. Red marks outside the heatmap: genes with outlier expression in the patient with HPV integration. Gene symbols: genes with outlier expression. Color of the gene symbols indicate their CERES score. Red symbols indicate genes with negative CERES scores, essential to tumor viability. Black symbols indicate genes not assessed by Project Achilles. **b** 134 TCGA CESC patients with 12 types integrated at 208 sites. **c** Number of genes upregulated (absolute $\log _2 \text {expression fold change} > 1$) in any of 160 patients from (**a**) and (**b**), where the gene’s CERES score < horizontal axis in at least one Project Achilles HPV^+^ cell line. Red: upregulated genes within 500 kbp of HPV integration sites. Blue: randomly chosen upregulated genes from the same patients. Blue data point: median of 100 permutations. Error bars: $\pm \text {SD}$ of 100 permutations. **d** Similar to (**c**), but instead the number of patients with at least one upregulated gene (absolute $\log _2$ expression fold change $> 1$), where the gene’s CERES score < horizontal axis in at least one Project Achilles HPV^+^ cell line

Having established that HPV integration results in changes in chromatin structure and dysregulated gene expression in cancer cell lines and patient tumors, we asked whether outlier expressed genes could play a driving role in tumorigenesis. We investigated the transcriptome of HPV^+^ HNSC and CESC tumors in TCGA. Out of the 71 HNSC HPV^+^ patients we examined, we found HPV integration sites in 22 of them by detecting transcribed chimeric sequences. Of these 22 patients, 16 (73%) displayed outlier expression of genes around HPV integration sites (Fig. 8a). Among 228 CESC patients, 139 had transcribed chimeric sequences and therefore HPV integration sites. Of those 139 patients, 85 (61%) tumors displayed outlier expression of genes around HPV integration sites (Fig. 8b).

Among the 5 cell lines, 26 HNSC tumors, and 85 CESC tumors, 231 genes in total showed outlier expression. HNSC patient TCGA-BA-5559, however, had an HPV integration at chr19:52,384,802, which disrupted the expression of 10 transcription factors with zinc finger domains (Fig. 8a). Many genes with outlier expression around HPV integration sites, such as *FOXA1* [47], *KLF12* [48], *SOX2* [49], *CUL2* [50], *CD274* [51], and *PBX1* [52], have previously reported roles in tumorigenesis.

Of 33 genes with outlier expression around HNSC tumor HPV integration sites, 11 harbored recurrent damaging mutations (missense, insertion, or deletion) among HPV^–^ HNSC tumors. These genes included histone deacetylase *HDAC4* (mutated in 6 tumors), receptor tyrosine kinase *ERBB2* (mutated in 6 tumors), transcription factor *SOX2* (mutated in 4 tumors), and transcription factor *NR4A2* (mutated in 3 tumors). This overlap did not show statistical significance at the population level (Fisher’s $p = 0.74$; odds ratio: 0.84), yet the involvement of these genes in progression of individual tumors remains possible.

We used g:Profiler [53] to identify dysregulated biological pathways more systematically (Additional file ﻿1: Table S3). Genes with outlier expression around HPV integration sites enriched for the GO terms “positive regulation of transcription by RNA polymerase II” (GO:0045944) and “cellular response to chemical stimulus” (GO:0070887, $q < 0.05$). Genes dysregulated in HNSC patients enriched for the GO term “positive regulation of nucleobase-containing compound metabolic process” (GO:0045935). Genes dysregulated in CESC patients enriched for “collagen catabolic process” (GO:0030574). These results suggest that genes upregulated upon HPV integration might activate transcription required for cellular proliferation, dysregulate cellular response to stress, anabolize nucleotides, and facilitate cellular invasion by degrading collagen within the basement membrane.

Project Achilles [54] provides CRISPR-Cas9 screening data on the essentiality of 18,333 genes for the viability of 625 cancer cell lines. This includes 4 HPV^+^ cell lines (SiHa, CaSki, SiSo [55], and SCC-152 [31]). These datasets report a CERES score for each gene, which quantifies its essentiality for cancer proliferation and survival [54]. Non-essential genes have a median CERES score of 0 and common core essential genes have a median CERES score of $-1$.

Among the 223 upregulated genes around the integration sites of 101 HPV^+^ patient tumors, 182 genes had negative CERES scores (Additional file 1: Table S4). For each patient, we performed 100 random permutations on the identity of the genes around their HPV integration sites, replacing them with other genes upregulated specifically in that patient (expression fold change $> 2$). Regardless of CERES score threshold used, we always found a higher number of upregulated essential genes in the original dataset than any of the permutation controls (Fig. 8c). Also, more patient tumors had at least one upregulated essential gene around their HPV integration site, compared to randomly selected upregulated genes (Fig. 8d).

## Discussion

Several hypotheses can explain how HPV integration promotes tumorigenesis. Integration induces the expression of E6 and E7 either through disruption of the viral DNA-binding protein E2 [56], disruption of untranslated regions of E6 and E7 [56], or the creation of stable viral-host fusion transcripts [57]. Alternatively, certain integration sites may become genomically unstable, facilitating aberrant chromosomal rearrangements [30], or may activate the expression of transposable elements, particularly short interspersed nuclear elements (SINEs) [58]. In many cases, transposable elements activate oncogenes and thereby initiate oncogenesis [59], a phenomenon termed onco-exaptation [60]. Onco-exaptation in endogenous retroviruses occurs through several mechanisms. These mechanisms include overexpression of long noncoding RNA (lncRNA) or protein-coding genes and also include production of chimeric or truncated proteins [60]. We propose that onco-exaptation of neighboring genes by HPV could also prove sufficient to drive oncogenesis. Consistent with prior reports [11, 30, 58, 61], our results point to a separate mechanism whereby HPV integration leads to altered expression and splicing of neighboring genes. Moreover, we identified active reorganization of local chromatin by CTCF binding to integrated HPV as a potential driver of local transcriptome dysregulation. HPV integration, as compared to only episomal HPV, may provide a selective advantage to tumor proliferation. Such an advantage would result in several phenomena seen in tumors with integrated HPV, including less favorable clinical outcomes [8], abundant detection of integration sites in HPV^+^ tumors, and even detection of multiple integration sites in some tumors.

In agreement with previous reports in HNSC tumors [11], our integrative analyses of a larger cohort including cell lines and patient samples across both HNSC and CESC suggest that HPV integration itself alters chromatin accessibility and the transcriptome, a previously under-appreciated phenomenon. These changes may contribute to tumorigenesis by upregulating the expression of neighboring genes, including some essential to tumor viability. In individual HPV integration sites, outlier expression of genes and changes in the epigenome occurred within 400 kbp of the integration. Examining integration sites in cell lines and patient tumors collectively uncovered significant chromatin, expression, and splicing differences within 100 kbp. Due to the integration of HPV at varying genomic regions, these analyses compared one sample to a population and therefore suffer from a lack of statistical power. We compensated for such limitations by using a restrictive definition for considering outlier genes and visualizing raw signal and summary statistics from individual samples obtained from multiple datasets.

We identified a possible role for CTCF binding to integrated HPV in dysregulating the host chromatin and transcriptome. A conserved CTCF binding site distinguishes tumorigenic and non-tumorigenic HPVs [12]. In episomal HPV, knockout of this binding site enhances the expression of the E6 and E7 oncogenes [13]. A distinct role of the binding site in integrated HPV resolves this apparent paradox and explains its recurrence in tumorigenic types.

Introduction of a new CTCF binding site by HPV may re-organize existing host topological domains. This can explain the extent of the changes in the chromatin and transcriptome seen here [14]. CTCF binding also plays a role in the life cycle of other DNA viruses, such as EBV, Kaposi’s sarcoma-associated herpesvirus, and herpes simplex virus 1 (HSV-1) [62, 63]. We showed here, however, EBV integration does not lead to significant changes in chromatin at integration sites—only HPV integration does. Our data agree with previous work showing that only some changes to CTCF binding sites alter chromosome conformation [64, 65]. In the case of hepatitis B virus, CTCF may bind both the episomal and chromatinized viral DNA and repress a specific enhancer crucial for viral transcription [66].

Recent studies have begun to shed light on how HPV integration can affect chromatin interactions [67, 68]. A recurrent HPV integration observed in 10 different patients results in formation of a new TAD [69]. Our observations in HeLa-S3 support this finding. Prior observations, however, have not linked the reorganization of the chromatin interactions to the conserved CTCF binding site of HPV as we propose here. Another important factor necessary for the formation of TAD boundaries, SMC1, also binds the HPV genome at its CTCF binding sites [70]. Our study suggests a link between CTCF binding within the host-integrated HPV and changes in the genome. Future confirmatory work could include genetic perturbation of the CTCF binding site within integrated HPV sequences to specifically measure its impact on chromatin interactions.

We showed that HPV integration can increase the expression of neighboring genes. We hypothesized that this, in turn, can predispose the host to tumor development. If true, the genomic position of the HPV integration site and the identity of its neighboring genes should matter. Otherwise, we would expect HPV found in cancers integrated into genomic regions without any neighboring oncogenes, since only a fraction of all genes can promote tumorigenesis. Reports on hotspot genomic regions in the host genome where HPV integrated [30, 58] and upregulated oncogenes around HPV integration sites [71] support the hypothesis of increased local expression. A recent study also suggests the preference of HPV integration sites for both FANCD2-associated genomic regions susceptible to tandem repeat formation and for enhancer-enriched genomic regions [72]. This provides more evidence for the presence of hotspot viral integration sites. The enrichment of HPV integration sites around genes [73] and transposable elements, especially SINEs [58], also supports this hypothesis.

If dysregulation of gene expression by HPV integration contributes to tumor development, we would expect to identify known oncogenes and master regulators of cancer-related pathways among the dysregulated genes in our analysis. Enrichment of these genes in growth-related pathways related to transcriptional regulation, nucleobase compound metabolism, and invasion-facilitating collagen catabolism confirm our expectation. Since upregulated transcripts do not necessarily prove change in biological pathways, we also investigated how knockout of these transcripts affects cellular viability. In agreement with recent studies [74], upregulated genes around HPV integration sites enriched among the most essential genes compared to upregulated genes distant from HPV integration sites. While we observed that some of the genes with outlier expression also harbor recurrent damaging mutations in HPV^–^ patients, this overlap did not show statistical significance at the population level (Fisher’s $p = 0.74$; odds ratio: 0.84). Nevertheless, the involvement of these genes in progression of individual tumors remains possible.

Most of the tumors we examined had chimeric transcripts that pinpointed integration sites. Only investigating these integration sites eliminated the possibility of detecting false-positive integration sites. This approach, however, can miss some true integration sites that do not produce a chimeric transcript. It will also miss sites where one read of a pair maps completely to the virus and the other completely to the host. Future studies using long-read whole-genome sequencing or targeted approaches such as Tagmentation-assisted Multiplex PCR Enrichment sequencing (TaME-seq) could identify HPV integration sites more exhaustively [75].

## Conclusion

Our results show that integration of HPV induces changes in local chromatin of the host and the local transcriptome. We predicted that these changes contribute to tumorigenesis. Our results suggest that interactions between integrated HPV chromatin and host chromatin trigger these changes, and that CTCF may play a key role in this process. Understanding the underlying mechanism of HPV–host chromatin interactions and their essentiality in tumorigenesis will better focus the future development of therapies for HPV^+^ cancers.

## Methods

### Multiple-testing correction

To control false discovery rate (FDR) over multiple comparisons, we used the Benjamini-Hochberg procedure [76] to attain *q*-values [77]. We used *q*-value cutoff of 0.05 unless we indicated another threshold in the manuscript.

### Genome assembly, annotations, and data processing

We generated a chimeric genome assembly and RefSeq gene transfer format (GTF) annotation of GRCh38 from Illumina iGenomes (https://support.illumina.com/sequencing/sequencing_software/igenome.html) and the National Center for Biotechnology Information (NCBI) RefSeq HPV16 K02718.1 assembly [78]. The resulting chimeric FASTA file had all the GRCh38 chromosomes, unplaced and unlocalized contigs, chrM (mitochondrial genome), EBV, and one additional chromosome containing the entire K02718.1 sequence. The GTF file contained all the Illumina iGenomes GRCh38 annotations and additional rows annotating K02718.1 coding sequences. For all experiments, we trimmed Illumina TruSeq adapters from FASTQ files with Trim Galore (version 0.4.4, https://www.bioinformatics.babraham.ac.uk/projects/trim_galore).

For CTCF ChIP-seq, input control ChIP-seq, and ATAC-seq, we used Bowtie2 [79] (version 2.2.6) with default parameters to align FASTQ files to the chimeric GRCh38-HPV16 genome. For RNA-seq, we used STAR (version 2.6.0c) [80], specifying options --outFilterMultimapNmax 2 --genomeSAindexNbases 6 --alignSJoverhangMin 8 --alignSJDBoverhangMin 4 --outFilterMismatchNoverReadLmax 0.05 to align the FASTQ files to the chimeric GRCh38-HPV16 genome.

### Cultured cell lines

We confirmed the identity of all cell lines via short tandem repeat profiling with the GenePrint 10 System (cat# B9510, Promega, Madison, WI) and performed mycoplasma testing prior to their utilization. We obtained 93-VU147T (RRID:CVCL_L895) as a kind gift from Bradly G. Wouters (University Health Network, Toronto, ON). We obtained SCC-090 (RRID:CVCL_1899) and SiHa (RRID:CVCL_0032) as kind gifts from Fei-Fei Liu (University Health Network, Toronto, ON). We obtained HMS-001 (RRID:CVCL_UH26) as a kind gift from James W. Rocco (The Ohio State University, Columbus, OH). We purchased CaSki (RRID:CVCL_1100) from the American Type Culture Collection (cat# CRL-1550, Manassas, VA).

We cultured 93-VU147T, SCC-090, SiHa, HMS-001 in Dulbecco’s modified Eagle medium (DMEM)/F12 [81] (Gibco, Waltham, MA) and CaSki in RPMI 1640 (Gibco, Waltham, MA). In all cases, we supplemented with 10% fetal bovine serum and 1% penicillin/streptomycin (cat# 450-201-EL, Wisent Bioproducts, Saint-Bruno, QC) and incubated in a humidified atmosphere containing 5% CO_2_ at 37 °C.

### RNA-seq

#### Library preparation and sequencing

We prepared samples for RNA-seq using the TruSeq Stranded Total RNA Sample Preparation kit with RiboZero Gold (Illumina, San Diego, CA). We performed RNA sequencing for each sample to ${\sim }80$ million paired-end 150 bp reads on an Illumina NextSeq 500 (Princess Margaret Genomics Centre, Toronto, ON). We collected input RNA using an AllPrep mini kit (Qiagen, Hilden, Germany).

#### Bioinformatics analysis

We used StringTie [82] (version 1.3.3b) to quantify TPM for genes in the chimeric GRCh38 annotation. We used DEXSeq (version 1.28.1) for alternative isoform analysis [83]. For DEXSeq, we downloaded Ensembl genes version 94 for compatibility with the DEXSeq protocol [84]. For each gene, we compared each sample against all the other samples. We repeated these steps for cell lines, HNSC, and CESC patients.

We generated a list of the exons with the most extreme difference in expression according to the DEXSeq negative binomial generalized linear model for all the genes around HPV integration sites. We quantified how the expression of each exon varies independent of the global expression of that gene [83]. For outlier exon expression, we again used a criterion of expression fold change $> 2$ compared to other cell lines:

2 $$\begin{aligned} \left\lvert \log _2 \frac{\text {exon count}_{\text {HPV}}}{\langle \text {exon count}_{\text {other}} \rangle }\right\rvert > 1 \text {.} \end{aligned}$$

Instead of using the SD cutoff, we corrected the *p*-values for multiple testing using the Benjamini-Hochberg method [76] and used a cutoff of $q < 0.2$ and minimum absolute fold change of 2 to select genes with alternative isoform expression.

#### Identifying HPV-induced outlier expression

To determine whether HPV integration significantly changed gene expression, we examined changes in transcription of individual genes, as measured in TPM. We used two criteria to identify outlier changes in gene expression which occurred due to HPV integration. First, we calculated expression fold change dividing $\log _2 \text {TPM}$ in the sample with HPV integrated at some locus ($\text{TPM}_{\text {HPV}^{+}}$) by median TPM in samples without HPV integrated at that locus ($\left\langle \text{TPM}_{\text {other}} \right\rangle$). For an HPV^+^ cell line, we only considered a gene an outlier if its expression fold change exceeded 2 (Additional file 1: Table S4).

This meant a $\log _2$ fold change greater than 1:

3 $$\begin{aligned} \left\lvert {\log _2 \frac{\text {TPM}_{\text {HPV}^{+}}}{\langle \text {TPM}_{\text {other}} \rangle }}\right\rvert > 1 \text {.} \end{aligned}$$

Fold change measurement, however, does not reflect dispersion in the expression of each gene. Second, therefore, we also required the difference in TPM to exceed at least twice the SD of TPM of that gene in other cell lines:

4 $$\begin{aligned} \left\lvert{\text {TPM}_{\text {HPV}^{+}} - \left\langle \text {TPM}_{\text {other}} \right\rangle }\right\rvert > 2 \text {SD} \end{aligned}$$

#### Empirical estimation of background distribution

To estimate the background distribution of genomic signal or outlier expression of genes at a given genomic region, we performed permutation analysis. To investigate how often outlier gene expression or alternative splicing occurred due to biological variation other than HPV integration, we permuted the sample identities of the HPV integration sites of each dataset multiple times. For TCGA data we permuted the sample data 100 times. For the 4 cell lines, we instead generated only 10 permutations. We did this to avoid over-representation of the effects from the cell types with fewer viral integrations.

In each dataset and in permutations, we examined the number of genes with expression fold change at several thresholds of expression or exon fold change separated by intervals of 0.25. We identified those genes with expression or splicing exceeding each threshold where the difference exceeded twice the SD.

To estimate the background distribution of allelic imbalance, we shuffled the cell type corresponding to each HPV integration. This matched each allelic imbalance value to a cell type with no HPV integration within the examined 500-kbp window. We performed 100 such permutations, and quantified the $\text {mean} \pm \text {SD}$ of this distribution. We counted the SNPs passing a cutoff on imbalance at a given distance from the HPV integration site.

We also investigated how the signal from RNA-seq, ATAC-seq, and CTCF ChIP-seq varies around samples with HPV integration sites compared to samples without HPV. At each integration site, we compared RPM from each assay for the cell line with HPV integration to the average in the other four cell lines.

We calculated RPM fold change (Eq. 1) for all 10-kbp genomic windows around any HPV integration site. We calculated the same measurement for 10 random permutations of HPV integration sites. For each permutation, we moved the location of each HPV integration site in each cell line to the location of a random integration site from another cell line, without replacement. We scrambled only the locations of the integration sites, leaving the assay data the same. For each assay, we examined the fold change of the original RPM against other cell types and compared with the fold change of the permuted RPM against other cell types. We did this for each 10-kbp window from the site of HPV integration up to 500 kbp away. Following the permutations, we conducted a two-sided *t*-test on the differences.

### CTCF ChIP-seq

#### Library preparation

We prepared 10 μL of both protein A and protein G beads through three washes of 5 mg/mL Dulbecco’s phosphate-buffered saline (dPBS) + bovine serum albumin (BSA). We added 10 µL of polyclonal CTCF antibody (cat# 2899, Lot 002, Cell Signalling Technology, Danvers, MA; RRID:AB_2086794) to the beads in 300 μL dPBS + BSA and left it to bind for >6 h of rotation at 4 °C. After incubation, we washed the beads three more times with dPBS + BSA. Then, we resuspended the beads in protease inhibitor (PI) and 100 μL of modified radioimmunoprecipitation assay buffer (RIPA): 10 mmol/L Tris-HCl, pH 8.0; 1 mmol/L EDTA; 140 mmol/L NaCl; 1% volume fraction Triton X-100; 0.1% mass fraction sodium dodecyl sulfate (SDS); 0.1% mass fraction sodium deoxycholate.

We trypsinized 1 million cells and then fixed for 10 min at room temperature in 300 µL of dPBS + 1% volume fraction formaldehyde. We added 15 µL of 2.5 mol/L glycine after fixing. Then, we washed the cells once in dPBS + PI before resuspending them in 300 µL of modified RIPA + PI. We sonicated the samples for 32 cycles of 30 s at full intensity using a Bioruptor Pico (Diagenode, Seraing, Belgium) and pelleted cell debris by spinning at $21130 \times \text{g}$ for 15 min. We set aside 15 µL of the supernatant as an input control and diluted the remaining supernatant with 1700 µL of modified RIPA + PI and 100 µL of washed beads. We incubated the samples at 4 °C overnight with rotation. We washed the beads with the following cold buffers in order: modified RIPA, modified RIPA + 500 µmol/L NaCl, LiCl buffer (10 mmol/L Tris-HCl, pH 8.0; 1 mmol/L EDTA; 250 mmol/L LiCl; 0.5% mass fraction NP-40; 0.5% mass fraction sodium deoxycholate), and finally twice with TE buffer (10 mmol/L Tris-HCl, pH 8.0; 1 mmol/L EDTA, ph 8.0). We resuspended the samples and inputs in 100 µL of de-crosslinking buffer (1% volume fraction SDS, 0.1 mol/L NaHCO_3_) and incubated at 65 °C for 6 h. We cleaned the samples and inputs using the Monarch PCR & DNA clean-up kit (New England BioLabs, Ipswich, MA), prepared libraries using the ThruPLEX DNA-seq Kit (Rubicon Genomics, Ann Arbor, MI), and size selected to 240–360 bp using a PippinHT 2% Agarose Cassette (Sage Science, Beverly, MA). For each sample, we sequenced three ChIP biological replicates and one input control to ${\sim }25$ million single-end 50 bp reads each on an Illumina HiSeq 2000 (Princess Margaret Genomics Core, Toronto, ON).

#### Bioinformatics analysis

We used MACS2 (version 2.1.2) software [85] to identify peaks and generate fragment pileup data using default parameters plus --nomodel --bdg, and using input as control. We also generated a log fold change enrichment bedGraph file by comparing fragment pileup to the input control lambda file generated by MACS2.

We used FastQC [86] (version 0.11.5) to assess the quality of ChIP-seq FASTQ files. After alignment with Bowtie2 and peak calling with MACS2, we used ChIPQC [87] (version 1.18.2) to assess enrichment quality. Input controls always had less than 0.7% fraction of reads in peaks, while ChIP experiments had an average of 9.4% fraction of reads in peaks (SD 6.4%). We merged the three replicates and found the following number of peaks passing a threshold of 5% FDR and 5-fold enrichment over input control: 32,748 in 93-VU147T, 22,353 in CaSki, 35,861 in HMS-001, 27,469 in SCC-090, and 37,161 in SiHa. We also assessed additional quality control metrics (Additional file 1: Tables S5–S7; https://doi.org/10.5281/zenodo.3780364[88]).

### ATAC-seq

#### Library preparation and sequencing

We assessed open chromatin using OMNI-ATAC [89] followed by size selection to 100–600 bp using a PippinHT 2% Agarose Cassette (Sage Science, Beverly, MA) and paired-end 125 bp sequencing on an Illumina HiSeq 2500 to a depth of ${\sim }60$ million reads per sample (Princess Margaret Genomics Core, Toronto, ON).

#### Bioinformatics analysis

We used MACS2 (version 2.1.2) software [85] to identify peaks and generate fragment pileup data using default parameters and --nomodel --shift -100 --extsize 200 --bdg --bampe. For analysis of ATAC-seq peaks, we used an FDR threshold of 5%.

To visualize the chromatin accessibility signal of multiple samples at HPV integration sites, we used the FPM measurement of each sample divided to the maximum FPM of that sample in the chromosome of HPV integration. This ensured all of the values ranged between 0 and 1 in that chromosome.

### Hi-C

We downloaded Hi-C data from the Encyclopedia of DNA Elements (ENCODE) [90] data portal [91] (HeLa-S3: ENCFF453NGH, ENCFF158BNB; PANC-1: ENCFF817XOP, ENCFF876LKL) and Gene Expression Omnibus (GEO) [92] (MCF-10A: GSE71862 [93]). To provide accompanying data, we downloaded ENCODE project bigWig files for HeLa-S3 CTCF ChIP-seq (ENCFF836JPY), HeLa-S3 reverse strand RNA-seq (ENCFF914DKK), HeLa-S3 forward strand RNA-seq (ENCFF585BBW), GM12878 reverse strand RNA-seq (ENCFF830QII), GM12878 forward strand RNA-seq (ENCFF470BSF), K562 reverse strand RNA-seq (ENCFF756LRF), K562 forward strand RNA-seq (ENCFF846MAT), H1-hESC reverse strand RNA-seq (ENCFF605VHG), H1-hESC forward strand RNA-seq (ENCFF094ZZR), PANC-1 CTCF ChIP-seq (ENCFF266BGZ), and PANC-1 RNA-seq (ENCFF142KMX).

For analysis of Hi-C data, we used HiC-Pro [94] (v2.11.4) with bin sizes of 20 kbp and 40 kbp and other default parameters. To compute TAD boundaries, we used the 40 kbp Hi-C matrices and hicFindTADs [95] (v3.7.2) --correctForMultipleTesting fdr and other default parameters. For plotting the TAD matrices, We used hicPlotTADs [95] (v3.7.2) with depth = 700000 and transform = log1p parameters.

### Allelic imbalance

We used dbSNP [46] (v147) to annotate polymorphisms around HPV integration sites. We defined allelic imbalance as the fraction of reads corresponding to the most-expressed allele. To avoid rare base-calling errors affecting the analysis, we only included SNPs with a minimum coverage of 10 reads and with at least 3 reads supporting each of the reference and the altered allele.

### TCGA datasets and analysis

#### RNA-seq datasets

We downloaded GRCh37-aligned TCGA RNA-seq datasets for 295 CESC patients and 547 HNSC patients [34]. We extracted FASTQ files from the binary alignment map (BAM) files using bam2fastq (https://gslweb.discoveryls.com/information/software/bam2fastq). We aligned the samples back to the chimeric GRCh38-HPV16 genome using STAR [80].

We used StringTie [82] to quantify TPM for each of the experiments according to the chimeric GTF annotation of GRCh38 and HPV16. From the available 547 HNSC patients, we identified 58 as HPV^+^. We identified all of the 295 CESC patients as HPV^+^. We used DEXSeq for alternative isoform analysis [83].

#### ATAC-seq datasets

For the 9 TCGA HNSC patients with ATAC-seq data, we downloaded GRCh38-aligned BAM files. We extracted FASTQ files from the BAM files using bam2fastq (version 1.1.0, https://gslweb.discoveryls.com/information/software/bam2fastq), trimmed adapters and low-quality sequencing reads from the FASTQ files with Trim Galore, and aligned the samples back to the chimeric GRCh38-HPV16 genome using Bowtie2 [79] (version 2.2.6). We used MACS2 (version 2.1.2) software [85] to identify peaks and generate fragment pileup data using default parameters and --nomodel --shift -100 --extsize 200 --bdg --bampe. For any analysis on ATAC-seq peaks, we used an FDR threshold of 5%.

### EBV^+^ lymphoblastoid cell line datasets

We investigated how the transcriptome and epigenome changed at the EBV integration sites of 4 lymphoblastoid cell lines: GM12873, GM12878, GM23248, and GM23338. Of these cell lines, ENCODE [90] supplies all 3 of total RNA-seq data, DNase-seq data, and CTCF ChIP-seq data for only GM12878 and GM23338. To provide 3 experiments for each assay, we added total RNA-seq and DNA-seq data from GM23248 and CTCF ChIP-seq from GM12873. For each of the 3 assays, this allowed us to compare potential differences arising from EBV integration in GM12878 to 2 other EBV^+^ lymphoblastoid cell lines.

### Identifying HPV types

For HNSC and CESC patients, we used the HPV type reported previously [21].

### Identifying HPV integration sites

We developed Polyidus to identify HPV integration sites with chimeric sequencing reads from any paired-end sequencing data. First, Polyidus aligns reads to a viral genome. It allows for partial mapping using local alignment and removes any sequencing fragment where neither read maps to the virus. Second, Polyidus aligns the selected reads to the host genome, permitting partial mapping. Third, Polyidus identifies *chimeric reads*: those reads mapped partially to the host genome and partially to the virus genome. Fourth, for each chimeric read, Polyidus reports the start and strand of integration in both the host and viral genomes. Polyidus also reports the number of chimeric reads supporting each integration site.

Polyidus finds highly confident integration sites which contain chimeric sequencing reads. Other methods perform the first two steps in reverse order [96], resulting in slower performance. While some previous methods also align to the virus first [97], either the software no longer appears available where specified at publication [98, 99], or they use BLAST [100, 101] instead of a faster short read aligner [102]. Unlike ViFi [58], Polyidus requires that the chimera match an existing viral genome reference. Polyidus does not use non-chimeric fragments where one read maps entirely to host and one read maps entirely to virus genome.

Polyidus uses Bowtie2 [79] (version 2.2.6) and vastly speeds up integration site finding. Polyidus identified integration sites at an average of 8 core-hours on a 2.6 GHz Intel Xeon E5-2650 v2 processor and 4 GB of RAM for whole genome sequencing data. Previous methods [103] require an average of 400 CPU core-hours.

We identified HPV integration sites in each sample using the sequence of the dominant HPV type in that sample. We excluded any HPV integration site found in more than 1 patient to avoid overestimation of outliers at potential hotspots of frequent integration [30]. In some cases, we found more than one HPV integration site in a 20-kbp window in one patient. Since we used RNA-seq for identifying our integration sites, some of these integration sites might occur as a result of splicing between the integrated HPV and neighboring host genomic regions. To avoid over-representing genomic regions with multiple integration sites, we only used the integration site with the highest number of chimeric sequencing reads.

### Motif enrichment analysis

We used FIMO (v4.11.2) [26] with the parameters --max-stored-scores 1000000000 --thresh 0.99 on a FASTA file containing the sequence of 189 HPV types (Additional file 1: Table S8) and the JASPAR 2016 [24] core vertebrate sequence motifs. We identified 5 sequence motifs with FIMO $q < 0.05$ among the 17 tumorigenic types reported in [21]. Using two-sample *t*-tests, we compared the FIMO enrichment scores of these motifs in the 17 tumorigenic types to 172 non-tumorigenic types, those types not reported among the [21] tumors.

## Supplementary Information

**Additional file 1:** Supplemental tables.

**Additional file 2:** Review history.

## Data Availability

Polyidus provides a framework to catch chimeric sequences using Python. It is available on GitHub (https://github.com/hoffmangroup/polyidus) [104] and deposited in Zenodo (https://doi.org/10.5281/zenodo.3780203) [105] under the GNU General Public License (GPL) version 3. We deposited our datasets for RNA-seq, ATAC-seq, and CTCF ChIP-seq of 5 HPV^+^ cell lines in the Gene Expression Omnibus (GEO) [92] (GEO accession: GSE143026) [106] and other processed data in Zenodo (https://doi.org/10.5281/zenodo.3780364) [88].
