# Supplementary material for: Human papillomavirus integration transforms chromatin to drive oncogenesis
Source: Genome Biol. 2023 Jun 27;24:142. doi: 10.1186/s13059-023-02926-9 (PMC10294493; doi:10.1186/s13059-023-02926-9)
Supplement: Supplementary file 2 — Additional file 2: Review history. [file 13059_2023_2926_MOESM2_ESM.docx]

**1^st^ round**

**Reviewer 1**

Summary

Karimzadeh et al. have delved into how HPV-mediated oncogenic transformation is caused by host chromatin re-organization caused by the introduction of new CTCF sites carried by the papillomavirus genome. They have taken a genomic approach to their studies, assessing the impact of CTCF binding in HPV-positive cancer cell lines. They discovered that chromatin within 100kb of the HPV integration site underwent a change in accessibility leading to the upregulation of genes associated with tumor viability. Based on these observations, they concluded that HPV-integration drives oncogenesis through local chromatin reorganization. Overall, this is an intriguing study, suggesting an interesting mechanism of HPV-induced oncogenesis which will likely be the focus of future work. The central model that the HPV genome serves as a carrier for novel CTCF binding sites is weakened by the absence of mechanistic data about CTCF binding in low-risk HPV's. The authors have also neglected to cite critical papers on HPV and host chromatin interactions, which must be addressed and contextualized relative to their findings (described below). This work falls within the scope of the Genome Biology journal, will be useful for the HPV community and should be accepted pending revisions.

Comments

1. Page 4 lines 33-36. The authors state "The consistent recruitment of CTCF at HPV integration sites in 4 different cell lines and altered ….plays a role in integration-dependent HPV tumorigenesis." This is a very strong statement and should be softened. In order to make this statement, that authors would have to provide a snapshot of the chromatin landscape in the same cells prior to HPV infection and integration.
2. Page 9 lines 5-11. It is commendable that the authors discuss the impact of EBV on the host genome and attempt to contextualize their findings in HPV. In this regard, they should cite Moquin et al., JV, 2018 and discuss how their findings compare with previously published observations. They should also discuss what the similarities and differences between EBV and HPV with the host nucleome might be since these are very different viruses. EBV has a lot more CTCF sites than HPV. Which ones are associated with integration?
3. Page 14 line 12. Please define the term onco-exaptation and explain why this is being used to describe HPV pathology rather than retrovirology. What are the similarities and what are the differences?
4. Page 14 lines 32-35. Please also cite the role of CTCF in regulating HBV expression published by D'Arienzo et al., Cellular Microbiology, 2021.
5. Page 15 line3. The authors claim: "[R]elated to transcriptional regulation, nucleobase compound metabolism and invasion-facilitated collagen catabolism". Are the cell lines being studied reported to have defects in these specific pathways in the literature? Changes in transcript levels are not necessarily indicative of changes in protein levels

**Reviewer 2**

Karimzadeh and collegues present here their work on HPV insertion and its impact on local chromatin and transcriptional changes using on both HPV cell lines and cancer samples. Overall, the paper is well written and describes promising observations made from distinct omics data (RNA-seq, ATAC-seq, ChIP-seq). However, the current manuscript could be improved by adding additional results/discussions on the functional consequences of these observations.
Please find below my detailed comments on the manuscript:

Major comments :
- 1/ My main concern is about the functional impact of the HPV CTCF site in regards with its role in the organization of the chromatin. According to the authors, the HPV (+CTCF site) integration can have strong functional impacts by modulating the local gene expression and chromatin landscape. As mentionned in the discussion, CTCF has a key role in the 3D genome organization and in the formation of TADs. So what is the impact of the introduction of this new CTCF site on the local chromatin organization at the TADs level ? I think that this point is crutial and must be discussed all along the manuscript. For instance, why the local effects are limited to 400kb around the HPV integration site ? Is it restricted to a single TAD ? In Figure 3, the HPV integration in the SiHa cell line has some effect upstream the insertion site ? Why only in the upstream region ? Any link to the TADs organization ? etc.
- 2/ The Figure 2 could lead to misleading interpretation. The panel a) shows the results of the motif analysis on all HPV subtypes. I would suggest to remove the log2 CTCF ChIP profile (pink) which is only from HPV16 and already shown in panel b) (whereas all the other results are from all strains).
Regarding panel b), the fact that "(…) the strongest CTCF ChIP-seq peak of the HPV genome aligned to the conserved CTCF sequence motif (...)" doesn't mean that CTCF really binds there. This is misleading, especially because the range of the y-axis is not the same on all the plots. Looking at the results, it seems that there is no CTCF peak on the SCC-090 and Caski cell lines, because the signal is clearly in the background compared to the Siha/93-VU1471 cell lines. An alternative would be to use the results of the peak calling itself, as the author mentionned that they used macs02 to call peaks in the ATAC-seq samples.

Minor comments :
1/ Data normalization. Most of the results presented in the manuscript are based on differences between log2 TPM (or FPM) fold-change. However, this type of normalization are intra-sample normalization and do not take into account composition bias between samples as for instance the TMM method (from the edgeR package). Please clarify this point.
2/ The authors describe that the HPV insertion has a transcriptional impact either downstream or upstream the integration site, but never in both direction. Is it related to the orientation of the HPV insertion itself ? Is there any other mechanisms which can explain this bias toward one direction ?
3/ In Figure 4, alteration in alternative splicing is only observed in the Caski cell line (but not in SCC-090 as mentioned in the text ?). Is it observed only locally around the HPV insertion site ? Is there any gene involved in splicing regulation which could be altered in this cell line ?
4/ According to Figure 4a, it seems that the up/downregulated genes are not necessarily the ones which are closest to the HPV insertion site. This observation seems a bit contradictory with the conclusion of Figure 5a where we do see a decrease of expression changes with the genomic distance. Please comment on these differences ?
5/ End of 2.4.1 "this represented a statistically insignificant difference". Please, reformulate. We cannot talk about statistically insignicant test in linear model. You can only state that your test was not significant.
6/ According to the manuscript, it seems that only upregulated genes have been used for GO analysis. Why not using all genes including down-regulated ones which could also be interesting ?
7/ Figure 7 nicely represents the changes at the expression level among several samples. What does "TPM Z-score" means ? Instead of showing 4 control samples, why not showing an average signal on a larger control cohort ?
8/ Discussion: "our results suggest that chromatin reorganization due to HPV integration occurs more frequently than previously appreciated". Please expound. The previous analyses showed HPV induces changes in chromatin and transcriptome. There was no mention of frequency. Otherwise, it would be better to rephrase this.
9/ Please detail the permutation strategy in the methods section
10/ Is the list of genes altered in HPV+ cells (2.3.1) available in sup Table 4 ? If it's correct, please add a mention in the text. Otherwise, it could be interesting to add this list in the sup data.

**Reviewer 3**

It is well established that human papillomavirus integration into host cellular chromatin can be a hallmark and a driver of HPV-mediated carcinogenic progression. Several studies based on TCGA and other data sets, plus molecular investigations, have clearly established that integration occurs in cancers caused by HPV infection and that it is more frequent in cervical cancer than in oropharyngeal cancer. Other reports have described a CTCF binding site present in the genome of several high-risk HPV. Here, the authors build on those findings, using several HPV-positive cancer cell lines in genome-wide analyses of CTCF binding, HPV integration status, and chromatin accessibility. Their findings complement ongoing work in the field and are a useful addition to the HPV integration literature, although the manuscript must do more to put their work in context with existing publications such as PMID 34848725. Their major advance is to combine studies on CTCF binding with the concept of HPV genome integration. Additional experimental approaches such as manipulating CTCF levels could also strengthen the investigation in the long term.

Specific comments:
1 - Rather than 'strains', distinct HPV are commonly referred to as genotypes.
2 - Although the abstract suggests that integration occurs only for high-risk HPV genotypes, there is evidence of low-risk HPV integration: see PMID 28040475
3 - The wording in section 2.1.1 could be improved with respect to which HPV are considered carcinogenic and which HPV were used in the authors' comparisons. Only some of the 17 genomes indicated in Figure 1A are typically considered to be definite carcinogens (PMID: 19350698), and the wording should be corrected accordingly. In 2.2.1, when the authors refer to the comparison of tumorigenic strains vs non-tumorigenic strains, are they referring to subsets of these 17 HPV genotypes, or to a comparison with other (low-risk or other) genotypes? The HPVs that were used in the comparison should be specified.
4 - Several of the cell lines used in the paper have been passaged extensively over decades of HPV research. The authors should include the source of cells used in the manuscript. In the future, it would be helpful to consider whether different isolates of a given cell line, for example SiHa cells, behave similarly in the authors' analyses.
5 - The comparison with EBV-containing cell lines is strong and adds to the paper.
6 - Section 2.5 refers to four HPV+ cell lines analyzed in Project Achilles, but Project Achilles analyzed many more HPV+ cell lines. Why were these four selected for analysis?
7 - The authors' model could be interpreted to predict that integration near certain genes important for carcinogenic progression drives the development of HPV-positive cancers. Considering the genes (or related pathways) identified as integration site outliers in HPV-positive OPSCC, are any of these frequently mutated in HPV-negative OPSCC?

**Reviewer 4**

Karimzadeh et al investigate changes in the transcriptome and epigenome of HPV-positive cell lines (HNSC and CESC) and TCGA HPV-positive tumors. They observe changes in chromatin state and expression of genes near the HVP integration sites identified in the host genome of these cell lines/tumors. They conclude that a CTCF motif present in the viral genome of HR-HPVs reorganizes the host chromatin and drives changes in host gene expression that are essential for oncogenesis.
Although the study conducted on 5 cell lines provides interesting new information, the analysis of TCGA tumor data is limited. One of the major issues is the interpretation of the causative role of the viral CTCF binding site brought in the vicinity of critical host genes in primary tumors, thus in vivo.

Main comments and questions

It is not clear from the introduction/discussion/cited literature: (i) whether integration is systematically seen in the most aggressive tumors, (ii) if not consistently detected in tumors, in what proportion is integration observed both in cervical and HN carcinomas and is there evidence showing that integration is correlated with aggressiveness, (iii) is integration a dynamic process and what about clonal expansion of cells with specific integration sites compared to cells that have episomal HPV or integration sites that do not perturb neighboring genes.

SiHa (lines 44 ->) "does not contain episomal HPV): is this a dynamic process, can integrrated HPV give rise to novel episomal forms i.e. via recombination and excision
Legend Fig. 1 b: "in each of the 5 cell lines" while only 4 are shown. In the fig, cell line 93-VU147T is said to have 7 IS but only one dotted line is indicated. Is the breakpoint in HPV identical for each of the 7 IS? The fig graphs do not match the text.

Section 2.2.1. in the TCGA dataset examined, only 9 tumors have concomitant RNA-seq and ATAC-seq data, and among these, only 1 (BA-A41H) show changes in chromatin accessibility and transcriptome. A full figure is dedicated to this single case.
Then, this single primary TCGA tumor is taken as a starting point for the next sections in which only cell lines are investigated (Figs. 3, 4 and 5). It is only in section 2.4.1 and Fig. 6 that primary tumors are considered.

Although the authors provide evidence that HPV integration causes cis-perturbation of host genes, the mechanism in vivo is not explored. Changes in host gene expression have been previously reported. The data obtained with primary tumors do not support a direct causative effect of a viral CTCF in vivo, the main novelty claimed by the authors. Experiments in which the CTCF binding site is mutated should be used to demonstrate causality.

Did consensus sequence search suggest the presence of an enhancer in the viral genome?

Read alignment on the reference genome: a better visualization may be obtained by aligning the RNA-seq (and ATAC-seq) reads on a hybrid custom genome in which HPV is integrated at the specific point revealed by RNA-seq (the breakpoints are known via chimeric reads).

Can the authors provide a quantification of the relative number of cells that have the HPV integration sites responsible for gene expression changes they observe? Is there a way of investigating clonal expansion (clone abundance) of these particular cells versus other clones that have episomal HPV or other 'non expanding' integration sites?

Bioinformatics (RNA-seq): how comparable are the data generated for cell lines by the authors to those available from TCGA in terms of read length and other specifications, and can they be used in the same pipeline without bias?

RNA-seq does not identify IS with great confidence as only reads that contain both viral and host sequences are considered, meaning that many integration sites may not be uncovered.

**Authors’ response**

Karimzadeh et al. have delved into how HPV-mediated oncogenic transformation is caused by host chromatin re-organization caused by the introduction of new CTCF sites carried by the papillomavirus genome. They have taken a genomic approach to their studies, assessing the impact of CTCF binding in HPV-positive cancer cell lines. They discovered that chromatin within 100kb of the HPV integration site underwent a change in accessibility leading to the upregulation of genes associated with tumor viability. Based on these observations, they concluded that HPV-integration drives oncogenesis through local chromatin reorganization.
Overall, this is an intriguing study, suggesting an interesting mechanism of HPV-induced oncogenesis which will likely be the focus of future work. The central model that the HPV genome serves as a carrier for novel CTCF binding sites is weakened by the absence of mechanistic data about CTCF binding in low-risk HPV's. The authors have also neglected to cite critical papers on HPV and host chromatin interactions, which must be addressed and contextualized relative to their findings (described below). This work falls within the scope of the Genome Biology journal, will be useful for the HPV community and should be accepted pending revisions.
We are pleased that the reviewer thought the paper should be accepted following revisions. We have worked to provide these revisions below.

Comments
1. Page 4 lines 33-36. The authors state "The consistent recruitment of CTCF at HPV integration sites in 4 different cell lines and altered ....plays a role in integration-dependent HPV tumorigenesis." This is a very strong statement and should be softened. In order to make this statement, that authors would have to provide a snapshot of the chromatin landscape in the same cells prior to HPV infection and integration.
We agree with the reviewer. We initially used the word “suggests” and understand why the reviewer proposes to further revise this statement.
To provide additional evidence for this suggestion, we added a new figure to the manuscript (new Figure 4), providing a snapshot of CTCF binding, topologically associated domain organization, and chromatin interaction around the HPV integration site of HeLa-S3 compared to other cells, further addressing the role for CTCF binding at HPV integration sites. To underscore that this is nonetheless not conclusive evidence, we also revised the statement in the main text of the manuscript to:
“The consistent recruitment of CTCF at HPV integration sites in 4 different cell lines and altered CTCF binding around integration sites suggest that CTCF might play a role in integration-dependent HPV tumourigenesis (Figure 3a, bottom).”

2. Page 9 lines 5-11. It is commendable that the authors discuss the impact of EBV on the host genome and attempt to contextualize their findings in HPV. In this regard, they should cite Moquin et al., JV, 2018 and discuss how their findings compare with previously published observations. They should also discuss what the similarities and differences between EBV and HPV with the host nucleome might be since these are very different viruses. EBV has a lot more CTCF sites than HPV. Which ones are associated with integration?
We thank the reviewer for suggesting this relevant manuscript. We added two new sentences to rationalize our comparison in the context of Moquin et al:

“Under this hypothesis, we expected that the integration of the 170 kbp Epstein-Barr virus (EBV) genome would fail to induce similar changes to the 8 kbp HPV genome. EBV, unlike HPV, shows a significant enrichment in association with gene-depleted chromosomes, often through the 2 kbp genomic region OriP [43]. OriP does not show a significant enrichment for cohesin CTCF binding sites, suggesting a potentially different mechanism of viral-host interaction than HPV [43].”

3. Page 14 line 12. Please define the term onco-exaptation and explain why this is being used to describe HPV pathology rather than retrovirology. What are the similarities and what are the differences?
We clarified the original definition of the term onco-exaptation and its scope among the endogenous retroviruses:
“In many cases, transposable elements activate oncogenes and thereby initiate oncogenesis [57], a phenomenon termed onco-exaptation [58]. Onco-exaptation in endogenous retroviruses occurs through various mechanisms, including over-expression of lncRNAs and proteincoding genes as well as production of chimeric or truncated proteins [58].”

4. Page 14 lines 32-35. Please also cite the role of CTCF in regulating HBV expression published by D'Arienzo et al., Cellular Microbiology, 2021.
We added a new sentence summarizing and citing this important study:
“In the case of Hepatitis B virus, CTCF may bind both the episomal and chromatinized viral DNA and repress a specific enhancer crucial for viral transcription [64].”

5. Page 15 line3. The authors claim: "[R]elated to transcriptional regulation, nucleobase compound metabolism and invasion-facilitated collagen catabolism". Are the cell lines being studied reported to have defects in these specific pathways in the literature? Changes in transcript levels are not necessarily indicative of changes in protein levels.
We agree with the reviewer that pathway analysis of upregulated transcripts does not prove change in protein levels and change in biological pathways. These pathways show enrichment in genes with dysregulated expression in multiple samples. We did this analysis to understand the possible biological pathways which might become dysregulated upon HPV integration. Therefore, we also looked into the closest possible simulation of the change in transcript level on cellular viability (gene essentiality score). We clarified this in a new sentence:
“Enrichment of these genes in growth-related pathways related to transcriptional regulation, nucleobase compound metabolism, and invasion-facilitating collagen catabolism, confirm our expectation. Since upregulated transcripts do not necessarily prove change in biological pathways, we also investigated how knock-out of these transcripts affects cellular viability. In agreement with recent studies [72], upregulated genes around HPV integration sites

enriched among the most essential genes compared to upregulated genes distant from HPV integration sites.”

Reviewer #2: Karimzadeh and collegues present here their work on HPV insertion and its impact on local chromatin and transcriptional changes using on both HPV cell lines and cancer samples. Overall, the paper is well written and describes promising observations made from distinct omics data (RNA-seq, ATAC-seq, ChIP-seq). However, the current manuscript could be improved by adding additional results/discussions on the functional consequences of these observations.
We thank the reviewer for their positive comments on the manuscript overall. Please see below for improvements in response to their specific comments.

Please find below my detailed comments on the manuscript:
Major comments :
- 1/ My main concern is about the functional impact of the HPV CTCF site in regards with its role in the organization of the chromatin. According to the authors, the HPV (+CTCF site) integration can have strong functional impacts by modulating the local gene expression and chromatin landscape. As mentionned in the discussion, CTCF has a key role in the 3D genome organization and in the formation of TADs. So what is the impact of the introduction of this new CTCF site on the local chromatin organization at the TADs level ? I think that this point is crutial and must be discussed all along the manuscript. For instance, why the local effects are limited to 400kb around the HPV integration site ? Is it restricted to a single TAD ? In Figure 3, the HPV integration in the SiHa cell line has some effect upstream the insertion site ? Why only in the upstream region ? Any link to the TADs organization ? etc.
We thank the reviewer for this insightful comment, which has motivated us to perform additional analyses. The HeLa-S3 cell line has both Hi-C data available and an HPV integration site. We compared HeLa-S3 Hi-C data to those of two other epithelial cell lines that lack an HPV integration site: pancreas (PANC-1) and breast (MCF-10A). New Figure 4 shows the presence of an HPV sequence is related to the inclusion of a TAD boundary missing in HPV– cancer cells. Specifically, the HPV integration in HeLa-S3 sits in a genomic region that splits a larger TAD observed in other epithelial cells. The HPV integration in HeLa-S3 also introduces a new CTCF peak in the host genome. This supports a CTCF-based model for the formation of this new TAD boundary.
We summarized these results as:
“To investigate the possibility of topologically associating domain (TAD) re-organization, we used the 3D Genome Browser³⁶ to compare TAD boundaries of HeLa-S3³⁷, a cervical carcinoma cell line with HPV integration, to other cell lines without HPV (PANC-1³⁸,

MCF-10A³⁹, GM12878, K562, and H1-hESC). HeLa-S3 cells contained chromatin interactions, TADs, and a TAD boundary not found in other cell types (Figure 4a–b). CTCF binding and RNA transcription increased in HeLa-S3 at the TAD boundary (Figure 4c). While little transcription occurred at this genomic region in PANC-1, K562, GM12878, and H1-hESC, strand-specific transcription data indicated more transcription from the reverse strand in HeLa-S3 (Figure 4c)”
In context of existing literature on this topic, other studies also report locus-specific TAD re-organization impacting a local genomic window, suggesting a strong genomic context to the biological impacts of HPV integration on upstream, downstream, and magnitude of changes in the transcriptome. This is discusses further in our discussion as:
“Recent studies have begun to shed light on how HPV integration can affect chromatin interactions [65,66]. A recurrent HPV integration observed in 10 different patients results in formation of a new TAD [66]. Our observations in HeLa-S3 support this finding. Prior observations, however, have not linked the reorganization of the chromatin interactions to the conserved CTCF binding site of HPV as we propose here. Another important factor necessary for the formation of TAD boundaries, SMC1, also binds the HPV genome at its CTCF binding sites [68]. Our study suggests a link between CTCF binding within the host-integrated HPV and changes in the genome, future confirmatory work could include genetic perturbation of the CTCF binding site within integrated HPV sequences to specifically measure its impact on chromatin interactions.”

- 2/ The Figure 2 could lead to misleading interpretation. The panel a) shows the results of the motif analysis on all HPV subtypes. I would suggest to remove the log2 CTCF ChIP profile (pink) which is only from HPV16 and already shown in panel b) (whereas all the other results are from all strains).
Regarding panel b), the fact that "(...) the strongest CTCF ChIP-seq peak of the HPV genome aligned to the conserved CTCF sequence motif (...)" doesn't mean that CTCF really binds there. This is misleading, especially because the range of the y-axis is not the same on all the plots. Looking at the results, it seems that there is no CTCF peak on the SCC-090 and Caski cell lines, because the signal is clearly in the background compared to the Siha/93-VU1471 cell lines. An alternative would be to use the results of the peak calling itself, as the author mentionned that they used macs02 to call peaks in the ATAC-seq samples.
As suggested by the reviewer, we removed the SiHa ChIP-seq signal within the HPV genome from Figure 2a and added MACS2 peak calling result of CTCF to panel b. Two of the four cell types, SiHa and 93-VU147T, had a peak called by CTCF. Given the fact that the signal is coming from multiple copies of HPV with different properties, we thought best to also keep the logFE signal. We clarified the fact that MACS2 did not find a CTCF peak in this region:
“MACS2 identified a CTCF ChIP-seq peak within the HPV genome of SiHa and 93-VU147T. In each of the other 4 cell lines these two cell lines, the strongest CTCF ChIP-seq peak of the HPV genome aligned to the conserved CTCF sequence motif described above.”

Minor comments :
1/ Data normalization. Most of the results presented in the manuscript are based on differences between log2 TPM (or FPM) fold-change. However, this type of normalization are intra-sample normalization and do not take into account composition bias between samples as for instance the TMM method (from the edgeR package). Please clarify this point.
Model-based analyses of RNA-seq data, such as those performed by edgeR, models variation at the gene and sample level for specific probability distributions. These models ideally require biological and technical variables for estimating their dispersion parameters. This is not possible when one of the comparison groups has only one sample, as is the case with our analysis. Due to this statistical limitation, throughout our manuscript, we tried to visualize sample-level signals obtained from various resources and using both raw signal and summary statistics. We further clarified this point in our discussion as:
“Due to the integration of HPV at different genomic regions, these analyses compare one sample to a population and therefore suffer from a lack of statistical power. We compensated for such limitations by using a restrictive definition for considering outlier genes and visualizing raw signal and summary statistics from individual samples obtained from multiple datasets.”

2/ The authors describe that the HPV insertion has a transcriptional impact either downstream or upstream the integration site, but never in both direction. Is it related to the orientation of the HPV insertion itself ? Is there any other mechanisms which can explain this bias toward one direction ?
We did not observe a significant association of the orientation of HPV integration and the direction of effects. We believe this depends on the genomic context of HPV integration site. Our analysis suggests the direction of transcribed chimeric sequence regulates the process, but we were under-powered to show a statistical significance:
“We investigated whether the direction of the chimeric HPV transcript affects the magnitude of changes in neighbouring genomic regions. Although the most highly upregulated genes occurred near the HPV integration sites that induced the chimeric transcript downstream of the integration site, the association did not pass the statistical significance cutoff (linear model 𝑝 = 0.21).”

3/ In Figure 4, alteration in alternative splicing is only observed in the Caski cell line (but not in SCC-090 as mentioned in the text ?). Is it observed only locally around the HPV insertion site ? Is there any gene involved in splicing regulation which could be altered in this cell line ?
Although among the cell lines we only observed HPV-neighbouring alternative splicing events around Caski (old Figure 4c, current figure 5c), we observed the occurrence of alternative

splicing events around HPV integration sites of both HNSC and CESC (old Figure 6b, current figure 7b) patients. Many upstream regulators show upregulation among all cancer types (https://doi.org/10.1016/j.omtn.2021.04.005). We looked into these pan-cancer upregulated RBPs, as well as all 1,085 genes linked to splicing regulation in gene ontology. We found that each of the 5 cell types show increased expression of many of these genes, but we did not observe anything specific to Caski to explain this at a global level. We added:
“Although perturbations in splicing regulators occur frequently in many cancer types [41], global dysregulation of splicing regulators within CaSki could not easily explain our findings. For example, the expression of genes involved in splicing regulation (16 biological processes containing the phrases “splicing”, “splite site”, or “spliceosome” in Gene Ontology (GO) v6.2) did not suggest a global change differentiating CaSki from the other 4 cell types. In addition, while 43/378 expressed genes involved in splicing had their highest expression in CaSki, other cell types also exhibited higher expression of other genes involved in splicing. Taken together, these results indicate that HPV integration can influence differential exon usage of neighbouring genes.”

4/ According to Figure 4a, it seems that the up/downregulated genes are not necessarily the ones which are closest to the HPV insertion site. This observation seems a bit contradictory with the conclusion of Figure 5a where we do see a decrease of expression changes with the genomic distance. Please comment on these differences ?
In almost all cases, we observed a clear change in transcription at the site of viral integration, where transcription of these genomic regions are silent in other cells occurs. As old Figure 5a (current Figure 6a) shows, however, individual genomic regions with extreme changes in transcription can occur hundreds of thousands of base pairs away from the integration site. The conclusion of old Figure 5 (current Figure 6) is that when we pool the data from multiple different cell types, we observe significant changes occurring within the first 100,000 bp, but individual changes in different samples occur within a larger genomic range. As the distance from the integration site increases, these extreme patterns are observed less. In our discussion, we reflect upon this point as:
“In individual HPV integration sites, outlier expression of genes and changes in the epigenome occurred within 400 kbp of the integration. Examining integration sites in cell lines and patient tumours collectively uncovered significant chromatin, expression, and splicing differences within 100 kbp.”

5/ End of 2.4.1 "this represented a statistically insignificant difference". Please, reformulate. We cannot talk about statistically insignicant test in linear model. You can only state that your test was not significant.
We corrected this sentence as:

“Although the most highly upregulated genes occurred near the HPV integration sites which induced the chimeric transcript downstream of the integration site, this represented a statistically insignificant difference the association did not pass the statistical significance cutoff (linear model p = 0.21).”

6/ According to the manuscript, it seems that only upregulated genes have been used for GO analysis. Why not using all genes including down-regulated ones which could also be interesting ?
We thank the reviewer for their suggestion. We intended to highlight the pathways affected by the activation of such genes. As the reviewer points out, however, changes in pathway activity may come from a combination of upregulated and downregulated genes as well. Therefore, we re-did this analysis including downregulated genes. Given the smaller number of downregulated genes, particularly among CESC patients, these results did not change. In Supplementary Table 3, we provided the results with only upregulated genes and all dysregulated genes for comparison, and in the text, we only refer to all dysregulated genes. In the text, we refer to “dysregulated” instead of upregulated genes for this analysis.

7/ Figure 7 nicely represents the changes at the expression level among several samples. What does "TPM Z-score" means ? Instead of showing 4 control samples, why not showing an average signal on a larger control cohort ?
Since different genes have a variable range of TPM values, we calculated the Z-score of TPM values within each gene among the 5 samples to better reflect the outlier samples for each gene. We clarified this in the figure caption as:
“Values show Z-score of TPM values of each gene among the 5 samples”
Showing the average expression within the figure would make the figure very complicated. To provide the requested information, we have instead added additional columns to Supplementary Table 4 which provides the TPM value in the sample with HPV integration, the average TPM value in all other samples of the same cohort, and standard deviation of TPM of that gene in all other samples of the same cohort.

8/ Discussion: "our results suggest that chromatin reorganization due to HPV integration occurs more frequently than previously appreciated". Please expound. The previous analyses showed HPV induces changes in chromatin and transcriptome. There was no mention of frequency. Otherwise, it would be better to rephrase this.
Some of the previous literature in this topic includes case reports, but another study (Kelley et al. 2017) showed the concept in a cohort of 72 HNSC tumours. We expanded on this previous report by using CESC in addition to HNSC and using a larger cohort while further studying the

concept using CTCF ChIP-seq and Hi-C experiments and datasets. We rephrased this section of our discussion as:

“In agreement with previous reports in HNSC tumours [11], our integrative analyses of a larger cohort including cell lines and patient samples across both HNSC and CESC suggest that HPV integration itself alters chromatin accessibility and the transcriptome, a previously under-appreciated phenomenon.”

9/ Please detail the permutation strategy in the methods section
We added a new subsection, “Empirical estimation of background distribution”, to our methods section describing the permutation analysis in more detail.

10/ Is the list of genes altered in HPV+ cells (2.3.1) available in sup Table 4 ? If it's correct, please add a mention in the text. Otherwise, it could be interesting to add this list in the sup data.
Yes, Supplementary Table 4 contains these genes. We provided a reference to this in section 2.3.1.

Reviewer #3: It is well established that human papillomavirus integration into host cellular chromatin can be a hallmark and a driver of HPV-mediated carcinogenic progression. Several studies based on TCGA and other data sets, plus molecular investigations, have clearly established that integration occurs in cancers caused by HPV infection and that it is more frequent in cervical cancer than in oropharyngeal cancer. Other reports have described a CTCF binding site present in the genome of several high-risk HPV. Here, the authors build on those findings, using several HPV-positive cancer cell lines in genome-wide analyses of CTCF binding, HPV integration status, and chromatin accessibility. Their findings complement ongoing work in the field and are a useful addition to the HPV integration literature, although the manuscript must do more to put their work in context with existing publications such as PMID 34848725. Their major advance is to combine studies on CTCF binding with the concept of HPV genome integration. Additional experimental approaches such as manipulating CTCF levels could also strengthen the investigation in the long term.
We thank the reviewer for their assessment of our work.
Thank you also for the suggestion to putting our work in context with Warburton et al. (PMID 34848725). We now include this work in Discussion while discussing the preference of HPVs for integrating at hotspot genomic regions. Additionally, we incorporated additional matched HiC and CTCF ChIP-seq data of HeLa-S3 to better support our claims.
“A recent study also suggests the preference of HPV integration sites for FANCD2-associated genomic regions susceptible to tandem repeat formation as well as enhancer-enriched genomic regions [Warburton et al.], providing more evidence for the presence of hotspot viral integration sites.”

Specific comments:
1 - Rather than 'strains', distinct HPV are commonly referred to as genotypes.
We understand the reviewer’s concern regarding the use of the word “strain”. The term genotype, however, also has the alternative meaning of genetic variations within the same HPV genome. Although terms such as genotype and strain have been used in the literature, we noticed that more recently the word type is becoming more common. Therefore, to avoid using the word strain and avoid other confusion, we switched to using the word “type”.

2 - Although the abstract suggests that integration occurs only for high-risk HPV genotypes, there is evidence of low-risk HPV integration: see PMID 28040475
We would like to emphasize the frequency of the integration. Some of the low-risk HPVs may also integrate (not as frequently) and result in tumours such as papillomas, as is the case with the low-risk HPV11 and formation of laryngeal papillomas referred to in the cited paper. To clarify this point, we cited the manuscript the reviewer mentions in the first sentence of the introduction, and we adjusted the abstract as:
“Frequent integration into the host genome occurs only for predominantly in tumourigenic types of HPV.”

3 - The wording in section 2.1.1 could be improved with respect to which HPV are considered carcinogenic and which HPV were used in the authors' comparisons. Only some of the 17 genomes indicated in Figure 1A are typically considered to be definite carcinogens (PMID: 19350698), and the wording should be corrected accordingly.
In 2.1.1, we had mentioned that these 17 strains originate from Campbell et al. reporting them among the TCGA HNSC and CESC tumours. We agree with the reviewer that just observing an HPV type in a tumour does not indicate its carcinogenicity. Therefore, we added new text to put these strains in context with the article the reviewer refers to:
“According to the evaluation criteria of the International Agency for Research on Cancer Monograph Working Group [22], among these 17 types (Figure 1a), 11 types show sufficient evidence for carcinogenicity. One type (HPV68b) shows limited evidence of carcinogenicity in humans but strong mechanistic evidence, three types (HPV26, HPV70, and HPV73) show limited evidence of carcinogenicity, and two types (HPV30 and HPV69) show phylogenetic analogy (belonging to the same type according to De Villiers et al. [23]) to HPVs with sufficient or limited evidence of carcinogenicity [24].“

In 2.2.1, when the authors refer to the comparison of tumorigenic strains vs non-tumorigenic strains, are they referring to subsets of these 17 HPV genotypes, or to a comparison with other (low-risk or other) genotypes? The HPVs that were used in the comparison should be specified.
We thank the reviewer for pointing out the missing information regarding this analysis. We added a new section to our methods (Motif enrichment analysis) and a new supplementary table 8, expanding on this analysis:
“We used FIMO (v4.11.2)27 with the parameters --max-stored-scores 1000000000 --thresh 0.99 on a FASTA file containing the sequence of 189 HPV strains (Supplementary Table 8) and the JASPAR 2016 core vertebrate sequence motifs. We identified 5 sequence motifs with FIMO 𝑞 < 0.05 among the 17 tumourigenic types reported in Campbell et al.²¹. Using two-sample t-tests, we compared the FIMO enrichment scores of these motifs in the 17 tumourigenic types to 172 non-tumourigenic types, those types not reported among the Campbell et al.²¹ tumours.”

4 - Several of the cell lines used in the paper have been passaged extensively over decades of HPV research. The authors should include the source of cells used in the manuscript. In the future, it would be helpful to consider whether different isolates of a given cell line, for example SiHa cells, behave similarly in the authors' analyses.
We agree with the reviewer that this is critical information to inform the readership and guide future experiments. To that end, we have added the following information to the Methods section:
Cultured cell lines
“We confirmed the identity of all cell lines via short tandem repeat profiling with the GenePrint 10 System (cat# B9510, Promega, Madison, WI), and performed mycoplasma testing prior to their utilization. We obtained 93-VU147T (RRID:CVCL_L895) as a kind gift from Bradly G. Wouters (University Health Network, Toronto, ON). We obtained SCC-090 (RRID:CVCL_1899) and SiHa (RRID:CVCL_0032) as kind gifts from Fei-Fei Liu (University Health Network, Toronto, ON). We obtained HMS-001 (RRID:CVCL_UH26) as a kind gift from James W. Rocco (The Ohio State University, Columbus, OH). We purchased CaSki (RRID:CVCL_1100) from the American Type Culture Collection (cat# CRL-1550, Manassas, VA). We cultured 93-VU147T, SCC-090, SiHa, HMS-001 in Dulbecco’s Modified Eagle Medium (DMEM)/F12⁷⁹ (Gibco, Waltham, MA) and CaSki in RPMI 1640 (Gibco, Waltham, MA). In all cases, we supplemented with 10% fetal bovine serum and 1% penicillin/streptomycin (cat# 450-201-EL, Wisent Bioproducts, Saint-Bruno, QC) and incubated in a humidified atmosphere containing 5% CO2 at 37 °C.”

5 - The comparison with EBV-containing cell lines is strong and adds to the paper.
Thank you for your positive comment on this analysis.

6 - Section 2.5 refers to four HPV+ cell lines analyzed in Project Achilles, but Project Achilles analyzed many more HPV+ cell lines. Why were these four selected for analysis?
Unfortunately, we were not able to find a systematic annotation of presence of HPV and whether HPV is integrated among the 625 cell lines in project Achilles. We restricted our analysis to cell lines where we had clear evidence of the presence of integrated HPV. Two of these cell lines, SiHa and Caski, were among our cohort of cell lines where we performed RNA-seq, ATAC-seq, and CTCF ChIP-seq on them. The other two cell lines have associated publications cited in our manuscript confirming the presence of integrated HPV: SISO (Sonoda et al. 1995) and SCC-1512 (White et al. 2007).

7 - The authors' model could be interpreted to predict that integration near certain genes important for carcinogenic progression drives the development of HPV-positive cancers. Considering the genes (or related pathways) identified as integration site outliers in HPV-positive OPSCC, are any of these frequently mutated in HPV-negative OPSCC?
We investigated whether transcriptionally dysregulated genes around HPV integration sites also contained somatic mutations among TCGA OPSCC patients. We found that 11/33 genes with outlier expression around HPV integration sites of HPV-positive OPSCC patients also have damaging mutations (missense, insertion, or deletion) in at least 2 HPV– patients. However, this overlap is not statistically significant. Regardless, we added a few sentences describing this analysis in our discussion:
“Of 33 genes with outlier expression around HNSC tumour HPV integration sites, 11 harboured recurrent damaging mutations (missense, insertion, or deletion) among HPV– HNSC tumours. These genes included histone deacetylase HDAC4 (mutated in 6 tumours), receptor tyrosine kinase ERBB2 (mutated in 6 tumours), transcription factor SOX2 (mutated in 4 tumours), and transcription factor NR4A2 (mutated in 3 tumours). This overlap did not show statistical significance at the population level (Fisher’s 𝑝 = 0.74, odds ratio: 0.84), yet the involvement of these genes in progression of individual tumours remains possible.“

Reviewer #4:
Karimzadeh et al investigate changes in the transcriptome and epigenome of HPV-positive cell lines (HNSC and CESC) and TCGA HPV-positive tumors. They observe changes in chromatin state and expression of genes near the HVP integration sites identified in the host genome of these cell lines/tumors. They conclude that a CTCF motif present in the viral genome of HR-HPVs reorganizes the host chromatin and drives changes in host gene expression that are essential for oncogenesis.

Although the study conducted on 5 cell lines provides interesting new information, the analysis of TCGA tumor data is limited. One of the major issues is the interpretation of the causative role of the viral CTCF binding site brought in the vicinity of critical host genes in primary tumors, thus in vivo.
We thank the reviewer for their constructive acknowledgement of the limitations of our work. Unfortunately, given the lack of epigenomic data, particularly CTCF ChIP-seq and Hi-C, for HPV+ TCGA samples, limits our ability to investigate the causality of viral CTCF binding site in TCGA data. In this revision, however, we added matched RNA-seq, CTCF ChIP-seq, and Hi-C data for HeLa-S3, which provides an example of re-organized TADs upon HPV integration. In addition, we investigated allelic imbalance to provide more evidence on our hypothesis that transcriptome changes occur on the chromosome containing the HPV integration sites. These new data and analyses are consistent with our model for the role of the CTCF binding site.

Main comments and questions
It is not clear from the introduction/discussion/cited literature: (i) whether integration is systematically seen in the most aggressive tumors, (ii) if not consistently detected in tumors, in what proportion is integration observed both in cervical and HN carcinomas and is there evidence showing that integration is correlated with aggressiveness, (iii) is integration a dynamic process and what about clonal expansion of cells with specific integration sites compared to cells that have episomal HPV or integration sites that do not perturb neighboring genes.

In our introduction, we indicate that 80% of invasive HPV+ tumours have HPV integration. A recent review article (https://doi.org/10.3390%2Fcancers13164089) discusses that HPV integration results in a less favourable outcome as well. We provide a citation to this article and added a new sentence in the introduction:
“Integration of HPV, therefore, results in a less favourable outcome [8].”
Given that many tumours exhibit multiple HPV integration sites, integration of HPV may provide a selective advantage. The mechanisms for these might include expression of viral oncoproteins, as well as disruption of host epigenome and transcriptome as we discuss. The first paragraph of our discussion reviews these mechanisms and the relevant literature:
“HPV integration, as compared to only episomal HPV, may provide a selective advantage to tumour proliferation. Such an advantage would result in several phenomena seen in tumours with integrated HPV, including less favorable clinical outcomes [8], abundant detection of integration sites in HPV+ tumours, and even detection of multiple integration sites in some tumours.”

SiHa (lines 44 ->) "does not contain episomal HPV): is this a dynamic process, can integrrated HPV give rise to novel episomal forms i.e. via recombination and excision

This might be possible, theoretically. Viral sequencing reads at the integration site were mostly chimeric, however, suggesting that the signal for SiHa is mainly from the integrated HPV. Legend Fig. 1 b: "in each of the 5 cell lines" while only 4 are shown. In the fig, cell line 93-VU147T is said to have 7 IS but only one dotted line is indicated. Is the breakpoint in HPV identical for each of the 7 IS? The fig graphs do not match the text.
We thank the reviewer for pointing out the mistake in referring to 5 cell types while 4 were shown. We have corrected that mistake.
With regard to the vertical lines in Figure 1b, they indicate the summit of the ATAC-seq peaks.
The host and viral integration positions are provided in Supplementary Table 1.

Section 2.2.1. in the TCGA dataset examined, only 9 tumors have concomitant RNA-seq and ATAC-seq data, and among these, only 1 (BA-A41H) show changes in chromatin accessibility and transcriptome. A full figure is dedicated to this single case. Then, this single primary TCGA tumor is taken as a starting point for the next sections in which only cell lines are investigated (Figs. 3, 4 and 5). It is only in section 2.4.1 and Fig. 6 that primary tumors are considered.
As the reviewer points out, the current format of the paper follows the following order:
1) finding an intriguing observation in public patient data
2) performing experiments and collecting data in cell lines available in our laboratory to validate and expand on the observation
3) use of more publicly available data for further validation
The limited number of patients with both RNA-seq and ATAC-seq data and HPV integration limited us in studying more patient samples in Figure 2, and as a result we performed experiments on patient-derived cell lines.

Although the authors provide evidence that HPV integration causes cis-perturbation of host genes, the mechanism in vivo is not explored. Changes in host gene expression have been previously reported. The data obtained with primary tumors do not support a direct causative effect of a viral CTCF in vivo, the main novelty claimed by the authors. Experiments in which the CTCF binding site is mutated should be used to demonstrate causality.
We agree with the reviewer on the limitations of our study. However, we think these limitations cannot be resolved with any single experiment and require further research outside the scope of this manuscript. For example, while using CRISPRi can methylate CTCF binding site, it would still not completely rule out other potential effects that DNA methylation may have on the expression of neighbouring genes. Similarly, CRISPR-Cas9 gene editing often results in unwanted deletions which confound the whole purpose of the experiment. The ultimate experiment would require transfecting HPV-negative squamous epithelial cell lines with HPV

and following them through time to select a subset where a complete HPV genome has integrated. This would be a major effort outside the scope of this manuscript.
To further clarify the limitation of our study, we added a new paragraph expanding on these limitations in our discussion and proposing future experiments:
“Recent studies have begun to shed light on how HPV integration can affect chromatin interactions⁶⁵, ⁶⁶. A recurrent HPV integration observed in 10 different patients results in formation of a new TAD⁶⁷. Our observations in HeLa-S3 support this finding. Prior observations, however, have not linked the reorganization of the chromatin interactions to the conserved CTCF binding site of HPV as we propose here. Another important factor necessary for the formation of TAD boundaries, SMC1, also binds the HPV genome at its CTCF binding sites⁶⁸. Our study suggests a link between CTCF binding within the host-integrated HPV and changes in the genome. Future confirmatory work could include genetic perturbation of the CTCF binding site within integrated HPV sequences to specifically measure its impact on chromatin interactions.”
In addition, we added a new analysis to investigate allele-specific expression, as quantified through allelic imbalance in our dataset. These results indicate that the changes in expression occur in the same chromosome that we observed the HPV integration. We summarized these results in the Figure 6c and the following paragraphs:
“To investigate whether these changes occurred in the same chromosome harboring integrated HPV sequences, we examined allele-specific expression close to the HPV integration site. If the HPV integration itself caused increased expression, we should observe higher allele-specific expression, as quantified by allelic imbalance. We defined allelic imbalance as the fraction of reads corresponding to the more-expressed allele for each SNP according to dbSNP v147⁴⁴. Allelic imbalance, therefore, ranges from 0.5 to 1. To avoid rare base-calling errors affecting the analysis, we only examined SNPs with a minimum coverage of 10 reads and at least 3 reads supporting each of the reference and altered alleles. Genes near integration sites had higher allele-specific expression. Higher allele-specific expression occurs particularly within 300 kbp of HPV integration in the sample with HPV integration, as compared to 100 permutations where we randomly assigned each allelic imbalance to a sample without HPV integration at that genomic position (Figure 6c). These results further confirm that downstream transcriptional changes occur on the same chromosome containing HPV sequence.”

Did consensus sequence search suggest the presence of an enhancer in the viral genome?
Our consensus sequence search identified a significant enrichment of CTCF binding sites among tumourigenic HPV strains, as opposed to non-tumourigenic HPV strains. Determining

whether these binding sites act as enhancers, however, requires the experiment we described above, and is out of scope for this manuscript.

Read alignment on the reference genome: a better visualization may be obtained by aligning the RNA-seq (and ATAC-seq) reads on a hybrid custom genome in which HPV is integrated at the specific point revealed by RNA-seq (the breakpoints are known via chimeric reads).
Since we do not have whole genome sequencing data for our cell lines and use RNA-seq reads for identifying the HPV integration site, we don’t know the full sequence from which we could construct the custom hybrid genome where both ends of the viral integration are clear. In Figure 3a, however, we added such a hybrid panel in the top right windows for SiHa. We included a gap in this genomic window at the end of the viral sequence to clarify the ambiguity in this custom hybrid genomic representation of the data.

Can the authors provide a quantification of the relative number of cells that have the HPV integration sites responsible for gene expression changes they observe? Is there a way of investigating clonal expansion (clone abundance) of these particular cells versus other clones that have episomal HPV or other 'non expanding' integration sites?
Quantifying the relative number of cells that have the HPV integration sites responsible for gene expression changes require single-cell RNA-seq data. Investigating clonal expansion in comparison to those with non-expanding integrations or episomal HPV requires another controlled experiment with proper labeling of these populations. Unfortunately, these experiments are outside the scope of this manuscript.
However, we added an analysis investigating the number of SNPs with allele imbalance with different thresholds and at different distances from the viral integration sites, which provides further information about the relationship between integration sites and gene expression changes. At a cutoff of 0.95 in allele imbalance within 100 kbp of the viral integration sites, we still observe 110 SNPs, which is still above the distribution observed in the background (Figure 6c). Allelic imbalance cutoff of 0.95 suggests at least 95% of the reads come from one source: the chromosome with HPV integration.

Bioinformatics (RNA-seq): how comparable are the data generated for cell lines by the authors to those available from TCGA in terms of read length and other specifications, and can they be used in the same pipeline without bias?
The datasets we investigated here are produced by different consortia. We applied the same bioinformatic pipeline to the raw data for all the datasets we used. However, to further avoid the variability among the datasets, we performed each analysis to the data coming from the same source. For example, our own cell line data were analyzed and compared together, the TCGA HNSC samples were compared to each other, and the TCGA CESC were compared to each other.

RNA-seq does not identify IS with great confidence as only reads that contain both viral and host sequences are considered, meaning that many integration sites may not be uncovered.
We agree with the reviewer. In our discussion, we point out this limitation. However, given that not all the samples in our cohort have available whole genome sequencing, we cannot fix this limitation.

**2^nd^ round**

**Reviewer 2**

The authors addressed all the previous points I raised. I think that the manuscript has been improved (especially with the chromatin structure part Fig4) and is now almost ready for publication.
I do not have any additional question.

**Reviewer 3**

The authors have carefully addressed my specific critiques and those of the other reviewers. Their data provide a detailed analysis and characterization of the consequences of HPV integration and the addition of a CTCF binding site in cellular chromatin. I am not convinced that the revisions have substantially addressed a main shared critique in the previous reviews, which was the need for functional studies on the role of CTCF binding in chromatin reorganization in HPV-positive cells.

**Reviewer 4**

I am pleased with the answers to my comments and to the concerns raised by reviewers 1-3. I particularly appreciate the new experiment carried out to examine allelic-specific expression. It is elegant and convincing (Fig. 6 c and associated paragraphs). Furthermore, the authors further comment on the limitations of their study by adding a paragraph in the discussion and proposing experiments that could address these limitations.
